# Emerging and Investigational Systemic Therapies in Recurrent/Metastatic Head and Neck Cancer After Progression on Immunotherapy

**DOI:** 10.3390/cancers17233817

**Published:** 2025-11-28

**Authors:** Freya F. Abraham, Ricklie Julian

**Affiliations:** 1 College of Medicine, University of Arizona, Tucson, AZ 85724, USA; freyaabraham@arizona.edu; 2 Cancer Center, University of Arizona, Tucson, AZ 85719, USA

**Keywords:** HNSCC, immunotherapy, recurrent/metastatic, targeted therapy, HPV, immunotherapy resistance

## Abstract

Background: Recurrent and metastatic head and neck squamous cell carcinoma (R/M HNSCC) after immune checkpoint inhibitor (ICI) progression represents a major clinical challenge. Between 60 and 80% of patients develop resistance, and historical salvage regimens like cytotoxic chemotherapy or chemotherapy plus cetuximab rarely extend median overall survival (mOS) beyond one year. Scope of Review: This review examines systemic therapies evaluated specifically in the post-ICI setting, emphasizing agents advancing to Phase II and III trials. Classes include chemotherapy combinations, ICI-based approaches, small-molecule targeted combinations, bispecific antibodies, antibody-drug conjugates (ADCs), and next-generation vaccines. Results: Promising signals have emerged across multiple therapeutic modalities. Targeted combination strategies have demonstrated encouraging response rates and survival outcomes in difficult-to-treat, PD-1-resistant disease. Antibody-based platforms, including antibody-drug conjugates and bispecific antibodies, continue to show consistent clinical activity across diverse patient populations, offering disease control and prolonged survival. Novel immunotherapies and therapeutic vaccines are also generating durable responses, particularly in biologically defined subgroups, highlighting the potential of immune-based precision treatments in R/M HNSCC. Conclusions: Comparative analysis highlights distinct advantages and limitations: chemotherapy ensures rapid shrinkage but poor durability; biomarker-driven small molecules achieve strong survival gains in narrow niches; ADCs and bispecifics offer balanced efficacy in unselected patients; and vaccine platforms deliver durable benefit in defined subsets. Together, these data signal a paradigm shift toward biomarker-guided, mechanism-driven strategies as the path to closing the post-ICI therapeutic gap in R/M HNSCC.

## Simple Summary

Patients with recurrent or metastatic head and neck squamous cell carcinoma who progress after immune checkpoint inhibitor therapy face limited treatment options, and conventional chemotherapy rarely achieves long-term control. This review synthesizes recent advances in systemic therapies developed for this setting, including targeted small-molecule combinations, antibody-based platforms, and novel immunotherapies or vaccines. Each therapeutic class offers distinct advantages, from targeted efficacy in biomarker-defined subgroups to broader activity across unselected populations. By comparing mechanisms of action, efficacy trends, and emerging clinical data, this review outlines how mechanism-driven and biomarker-guided strategies are reshaping the post-immunotherapy treatment landscape and moving the field toward more durable and personalized outcomes.

## 1. Introduction

Recurrent and metastatic head and neck squamous cell carcinoma (HNSCC) remains a major therapeutic challenge. Approximately half of patients with locally advanced disease develop recurrence or metastasis despite multimodality curative-intent therapy [1]. Among those with advanced disease, 60–80% progress after first-line immune checkpoint inhibitor (ICI)-based therapy, reflecting both primary and acquired resistance [2]. Historically, second-line systemic therapies in the pre-ICI era achieved poor outcomes, with a median progression-free survival (PFS) of 2.2 months and overall survival (OS) of 6.1 months. After progression on PD-1/PD-L1 inhibitors, there is no universally accepted standard of care; treatment often defaults to palliative chemotherapy or chemotherapy plus cetuximab, regimens that achieve modest and transient efficacy [3].

The introduction of ICIs such as pembrolizumab and nivolumab transformed the recurrent/metastatic landscape. ICIs restore antitumor immune responses by blocking inhibitory pathways that suppress T-cell activation. In HNSCC, the PD-1/PD-L1 axis is the principal therapeutic target. PD-1 on activated T cells binds PD-L1 on tumor or immune cells to dampen cytotoxic activity. Monoclonal antibodies that block PD-1 (pembrolizumab, nivolumab) or PD-L1 (durvalumab, atezolizumab) reverse this suppression and enhance antitumor immunity. A second inhibitory receptor, CTLA-4, regulates early T-cell activation and is targeted by agents such as ipilimumab, though its role in HNSCC remains less established. The KEYNOTE-048 trial established pembrolizumab monotherapy for PD-L1 CPS ≥1 and pembrolizumab plus platinum/5-FU chemotherapy for all comers as standards of care [4]. These regimens demonstrated superior OS compared with the EXTREME regimen (cetuximab plus platinum/5-FU), with generally better tolerability and fewer grade ≥ 3 toxicities [4]. Landmark trials such as CheckMate 141 and KEYNOTE-040 reinforced these findings, leading to global guideline adoption [5].

Despite these advances, ICI failure remains an unresolved problem [6]. Conventional salvage regimens (taxanes, methotrexate, cetuximab) yield response rates of 21–69% but with short median PFS (2.9–5.5 months) and OS (6.3–13.3 months) [7,8]. Although prior ICI exposure may enhance chemotherapy responsiveness, no novel agent has yet demonstrated Phase III superiority. As a result, there is no clear standard of care choice in second-line treatment [9,10]. The rapid integration of ICIs into first-line therapy has created a growing population of patients with ICI-refractory HNSCC, an unmet need likely to expand as ICIs move into curative-intent settings (KEYNOTE-689, NIVOPOSTOP GORTEC 2018-01) [11,12]. Accordingly, this review focuses on systemic therapies evaluated specifically after ICI failure, with emphasis on agents advancing into Phase II and III clinical trials. We synthesize evidence across novel therapeutic classes—including ADCS, bispecific antibodies, ICI combinations, and molecularly targeted therapies—to assess their efficiency, safety, and potential to redefine the post-ICI landscape.

## 2. From Guidelines to Chemotherapy Combinations: Benchmark and Novel Strategies

After progression on immunotherapy, treatment options for R/M HNSCC generally revert to cytotoxic chemotherapy or EGFR-targeted agents [13,14,15]. Recommended regimens include single-agent taxanes, methotrexate, capecitabine, or cetuximab [13,14,15]. Among these, cetuximab—an EGFR-targeting monoclonal antibody—historically achieved ORRs of only 10–13% and OS of 5–6 months in pre-ICI studies [13,14,15]. More recent post-ICI analyses, including INTERLINK-1 (ORR 23.9% in the placebo/cetuximab arm) and institutional series, suggest variable but occasionally higher activity, possibly reflecting immune priming [16]. Nonetheless, efficacy remains limited, and cetuximab monotherapy now functions primarily as a benchmark comparator for newer agents rather than a definitive salvage standard [13,14,15]. A dedicated Phase II trial (NCT04375384) is underway to clarify its post-ICI role [17].

To improve outcomes, chemotherapy backbones have been reintroduced—most notably paclitaxel plus cetuximab. This regimen leverages the direct anti-EGFR and immune-mediated activity of cetuximab, the cytotoxicity of paclitaxel, and potential immune-priming effects from prior ICI exposure [13,14,15]. Clinical outcomes from prospective Phase II trials are compelling: the Japanese jRCTs051200040 study reported ORR 69.6%, DCR 93.7%, and median OS 13.3 months, although only one enrolled patient was p16-positive, limiting conclusions in HPV-associated disease [7]. The international PACE-ACE trial achieved ORR 47.4% and median OS 14.0 months, while also having limited representation of p16-positive patients [18]. Expected chemotherapy-related toxicities were frequent, with ≥grade 3 AEs in approximately 65%, primarily neutropenia, infection, and neuropathy [18]. However, the efficacy positions paclitaxel plus cetuximab as the most reliable salvage benchmark and the standard comparator for ongoing trials of novel agents in the post-ICI setting.

This review focuses on answering the question of what options HNSCC patients have after failure on immunotherapy by examining data from novel therapies. Clinical data suggest that prior ICI exposure may “prime” tumors for chemotherapy, resulting in higher response rates than historically observed, which underscores sequencing as a critical therapeutic lever [6]. Next-generation trials are investigating paclitaxel-based regimens, both with established partners like cetuximab and with novel targeted agents, to capitalize on this immune priming effect and extend survival benefits in the post-ICI setting [13,14,15].

### 2.1. FID-007 (Nanoparticle Paclitaxel)

Investigators are now exploring next-generation chemotherapy platforms such as nanoparticle formulations, including FID-007, which aim to preserve the efficacy of taxane-based therapy while improving delivery and potentially reducing systemic toxicity [19]. FID-007 is a novel chemotherapy platform that represents a significant innovation in the delivery of paclitaxel for R/M HNSCC [19]. Unlike conventional formulations, FID-007 uses a polyethyloxazoline (PEOX) polymer excipient to encapsulate paclitaxel [19]. This design improves pharmacokinetics and biodistribution, allowing the drug to remain soluble until it enters tumor cells [19]. Because HNSCC tumors often exhibit leaky vasculature, FID-007 preferentially accumulates at the tumor site, enabling enhanced delivery at lower or comparable doses relative to existing taxane formulations [19]. Preclinical studies confirmed that FID-007 was more effective than nab-paclitaxel at lower or comparable taxane doses, supporting its advancement into clinical evaluation [19]. The first in-human Phase I trial (NCT03537690) determined the recommended Phase II dose (RP2D) to be 125 mg/m^2^ [19]. A total of 46 patients with advanced solid tumors were enrolled, including 9 patients with heavily pretreated HNSCC, all of whom had received prior immune checkpoint inhibitors [19]. Treatment was administered on Days 1, 8, and 15 of a 28-day cycle [19]. The most common all-grade treatment-related adverse events (TRAEs) included rash (72%), alopecia (52%), leukopenia (46%), pruritus (43%), and neutropenia (41%) [19]. Grade 3–4 toxicities consisted primarily of maculopapular rash (35%), neutropenia (20%), and leukopenia (20%) [19]. Notably, the drug’s mechanism of action was designed to mitigate peripheral neuropathy, which is a leading and dose-limiting toxicity of taxanes [19]. Phase I results support this, as no high-grade neuropathy was observed [19]. Efficacy in the HNSCC cohort was notable. The ORR reached 56% (5 out of 9 patients achieved partial responses) [19]. Historical ORRs for taxane monotherapy in this population typically range from 14% to 27% [19]. Importantly, three of the five responding HNSCC patients had previously received taxane-based therapy, suggesting that FID-007 may overcome acquired resistance to conventional taxanes [19]. These findings highlight the potential of FID-007 not only as a reformulation but as a therapeutic advance with unique clinical activity in the post-ICI setting. Based on these encouraging results, a Phase II study (NCT06332092) is planned to test FID-007 in combination with cetuximab, leveraging both its improved delivery profile and the immunogenic effects of EGFR blockade [20].

### 2.2. SI-B001 (Izalontamab) Plus Paclitaxel

While novel formulations like FID-007 aim to optimize the delivery and tolerability of paclitaxel, another strategy is to pair the chemotherapy backbone with next-generation biologics designed to address resistance mechanisms that limit the durability of EGFR inhibition [21]. One key pathway involves HER3 (ERBB3), an ErbB family receptor that lacks intrinsic kinase activity but forms heterodimers with EGFR and ERBB2, activating proliferative and survival signaling cascades [21]. Bispecific antibodies targeting both EGFR and HER3 are therefore being developed to achieve deeper and more durable receptor blockade [21]. Izalontamab (SI-B001) represents one such agent, and when combined with paclitaxel, it leverages the established cytotoxic efficacy of taxanes while simultaneously expanding the mechanism of action through dual receptor inhibition [22]. This approach aims to overcome resistance to single-agent EGFR blockade [22].

Preclinical studies demonstrated potent antitumor efficacy, with the combination of SI-B001 and paclitaxel (with or without carboplatin) producing synergistic activity and outperforming cetuximab-based combinations in xenograft models [23]. These findings provided the rationale for clinical development in R/M HNSCC following progression on PD-1 inhibitors. Initial Phase I dose-escalation studies established that SI-B001 monotherapy was well tolerated, with an RP2D ranging from 9 to 16 mg/kg weekly. The most common TRAEs were rash (42%) and paronychia (25%), consistent with EGFR-inhibitor class effects but with generally low frequencies of grade ≥ 3 adverse events. In terms of early efficacy, the Phase I study, which included 3 HNSCC patients, observed one confirmed partial response (PR), with the patient continuing treatment for nine months. Building on this, the Phase II S206 trial in 25 patients further evaluated SI-B001 in combinations with chemotherapy, where the combination with paclitaxel (Group A) yielded a highly encouraging ORR of 64.3% and a median progression-free survival (mPFS) of 5.6 months in patients who had received ≤2 prior lines of therapy and failed immunotherapy [23]. This robust response rate compares favorably to historical controls, including taxane-cetuximab regimens, highlighting the potential added value of dual EGFR/HER3 blockade in this population [23]. By contrast, SI-B001 monotherapy (S209 trial) in heavily pretreated patients demonstrated only modest activity (ORR 22.2%, mPFS 2.7 months), underscoring the synergy achieved with taxanes [23]. Toxicity with the combination was manageable, with grade ≥ 3 treatment-related hypomagnesaemia observed in the monotherapy cohort (9%) and rash/anemia/leukopenia seen in the combination cohort (up to 16%) [23]. These promising Phase II data have positioned SI-B001 as an emerging therapeutic backbone.

## 3. Next-Generation Immunotherapy Combinations

While synergistic combinations leveraging chemotherapy and targeted agents show promise, a concurrent strategy involves developing novel agents that directly modulate the immunosuppressive tumor microenvironment (TME) to restore anti-PD-1 efficacy. A key mechanism of resistance involves the Transforming Growth Factor-beta (TGF-*β*) pathway, a crucial regulator that inhibits T cell proliferation, promotes immunosuppressive cells, and facilitates immune evasion [24]. This realization has driven the development of bifunctional fusion proteins designed to block both PD-L1 and TGF-*β* signaling simultaneously.

### 3.1. Retlirafusp Alfa (SHR-1701)

Retlirafusp alfa (SHR-1701) is a bifunctional fusion protein that simultaneously blocks PD-L1 and traps TGF-β through its extracellular TGF-β receptor II domain, thereby aiming to restore anti-tumor immunity by reversing T-cell exclusion and dampening immunosuppressive signaling in the TME [25]. Preclinical studies supported this approach by showing that dual inhibition of PD-L1 and TGF-β fosters an inflamed tumor phenotype, enhances antigen presentation, and augments infiltration and activation of cytotoxic T cells and NK cells [25]. This provided a rationale for combining SHR-1701 with cytotoxic backbones such as carboplatin and nab-paclitaxel [26].

In an early Phase I trial across advanced solid tumors, SHR-1701 demonstrated tolerability, establishing 30 mg/kg every three weeks as the recommended Phase II dose. Preliminary responses were noted in HNSCC, with an ORR of 20.0% in a heavily pretreated HNSCC cohort [25]. Building on this foundation, a prospective single-arm Phase II study investigated the regimen of SHR-1701 (30 mg/kg, every three weeks) plus nab-paclitaxel and carboplatin in 12 patients with pretreated R/M HNSCC (ChiCTR2300070675) [26]. Despite the smaller sample size, the combination demonstrated promising anti-tumor efficacy and manageable toxicities. Grade 3–4 adverse events occurred in 4 (33.33%) patients. The most common of these were hematologic: white blood cell count decreased (25.0%), neutrophil count decreased (16.67%), and anemia (16.67%) [26].

### 3.2. FLX475 (Tivumecirnon) Plus Pembrolizumab

Other novel combinations employ complementary approaches focused on preventing the infiltration of inhibitory immune cell populations. The combination of FLX45 (tivumecirnon), a potent and selective CCR4 antagonist, and pembrolizumab represents a novel strategy to overcome ICI resistance by targeting the TME [27]. Mechanistically, FLX475 blocks CCR4, preventing the recruitment of immunosuppressive regulatory T cells (Tregs) into the tumor and thereby reducing local immunosuppression while augmenting the activity of cytotoxic CD8+ T cells [27]. This concept was supported by preclinical studies demonstrating that CCR4 blockade enhances PD-1/PD-L1 responsiveness and provided the rationale for combining FLX45 with pembrolizumab [27]. Clinical activity was evaluated in the Phase II portion of the FLX475-02 trial (NCT03674567), which enrolled checkpoint inhibitor-experienced patients with R/M HNSCC [28]. Among 32 evaluable patients who had received a median of three prior systemic regimens, the ORR was 15.6% (95% Cl 6–32%), with confirmed partial responses in five subjects [28]. Efficacy appeared enriched in biomarker-defined subgroups, with an ORR of 18.2% in patients with a PD-L1 combined positive score (CPS) ≥1 and an ORR of 22.2% (95% Cl 9–46%) in the HPV-positive cohort [28]. The treatment was generally well tolerated, with the only clearly FLX475-related adverse event being asymptomatic and reversible QT prolongation, which was managed with dose reduction [28]. These findings suggest that while overall activity was modest, the enrichment of responses in HPV-positive tumors supports further investigation of this combination in checkpoint inhibitor-experienced HNSCC [28].

## 4. Targeted Therapies: Small Molecule Inhibitors

Targeted therapies are designed to inhibit specific molecular pathways that drive tumor growth and survival. For patients with R/M HNSCC who have progressed on standard immunotherapy, these agents represent a critical area of investigation. They can be broadly categorized into small molecule inhibitors, which target intracellular pathways, and larger antibody-based biologics that target cell surface proteins. Small molecule inhibitors are typically orally administered drugs that can penetrate the cell membrane to interfere with intracellular signaling cascades involved in cell proliferation, survival, and DNA repair.

### 4.1. Targeting CDK4/6 with Dalpiciclib and Palbociclib

Cell cycle dysregulation is a hallmark of HPV-unrelated HNSCC, frequently driven by inactivation of p16^INK4A and overexpression of cyclin D1, which together lead to hyperactivation of cyclin-dependent kinases 4 and 6 (CDK4/6) [29,30]. Genomic alterations in CDKN2A and CCND1 occur in approximately 58% and 31% of HPV-unrelated cases, respectively [31]. This dysregulation facilitates unchecked G1-S phase progression, promoting tumor proliferation and resistance to therapies such as EGFR inhibitors [32]. In HPV-unrelated HNSCC, RB inactivation typically arises through hyperactivation of the CDK4/cyclin D complex [33,34,35]. CDK4/6 inhibitors are designed to arrest cell cycle progression by blocking this pathway [29]. When combined with EGFR-targeted therapies such as cetuximab, they may counteract resistance by suppressing deregulated cyclin D1 signaling [36,37]. Preclinical studies support this rationale: selective CDK4/6 inhibition induced G1 arrest and synergistically reduced tumor cell viability when combined with EGFR blockade in HPV-unrelated HNSCC cell lines [36,37].

Dalpiciclib plus cetuximab has recently emerged as a promising strategy for patients with anti-PD-1 resistant, HPV-negative R/M HNSCC [38,39]. Dalpiciclib is an oral small-molecule CDK4/6 inhibitor that, when combined with cetuximab, provides dual targeting of both EGFR and the cell cycle [38,39]. This strategy was specifically designed to address the clinical challenge of resistance to anti-PD-1 agents, which are standard first-line therapy for HNSCC, by introducing a targeted combination for patients who have already progressed on immunotherapy [38,39]. In a Phase II trial (NCT05721443) enrolling 28 patients resistant to PD-1 therapy and cetuximab-naïve, the regimen demonstrated a highly encouraging ORR of 67.9% (95% Cl, 49.0–82.0) and a disease control rate (DCR) of 89.3%. mPFS was 5.3 months, and median overall survival (OS) was 17.0 months [38,39]. The safety profile was consistent with other CDK4/6 inhibitor regimens, with manageable toxicities: grade 3 neutropenia and leukopenia each occurred in 32.1% of patients, but no grade 4–5 TRAEs were reported [38,39]. These outcomes compare favorably to historical post-ICI benchmarks (ORR 6–19%, mOS 5.3–8.9 months), underscoring the potential of this combination as a meaningful advance for a biomarker-selected subset of HNSCC [38,39].

Palbociclib plus cetuximab represents another major CDK4/6-based strategy. Like dalpiciclib, palbociclib selectively inhibits CDK4/6, preventing G1-S progression and thereby sensitizing tumors to EGFR inhibition [36,37]. Preclinical studies demonstrated synergy in HPV-negative HNSCC, especially in tumors with CDKN2A alterations or cyclin D1 amplification [40]. While palbociclib monotherapy in heavily retreated patients with CDKN2A-altered head and neck cancer met the pre-specified criteria for a signal of activity, the ORR was limited at 4% and the DCR was 40%, indicating limited utility as a single agent [41]. Early phase I trials established the feasibility of palbociclib at 125 mg/day (days 1–21 of 28-day cycles) combined with weekly cetuximab, showing disease control in 89% of patients, including partial responses in 22%, even among cetuximab and platinum-resistant cases [42]. Toxicity was manageable, with reversible neutropenia as the most frequent adverse event; non-hematologic events such as rash and hypomagnesemia reflected cetuximab’s class profile [42]. In Phase II studies in 32 cetuximab-resistant patients, ORR was 19%, whereas in HPV-related, cetuximab-resistant oropharyngeal cancer, efficacy was minimal (ORR 4%) [43,44]. However, the randomized Phase II PALATINUS trial (NCT02499120) failed to demonstrate a survival benefit in biomarker-unselected disease: median OS was 9.7 months with the combinations versus 7.8 months with cetuximab alone (HR, 0.82; 95% Cl, 0.54–1.25; *p* = 0.18) [45]. Importantly, exploratory biomarker analyses indicated a potential OS advantage in CDKN2A-altered tumors, with an HR of 0.38 and median OS of 9.7 versus 4.6 months [45]. This biomarker signal provided the rationale for the ongoing Phase III trial (NCT04966481), which specifically enrolled HPV-negative, CDKN2A-altered, anti-PD-1-resistant patients to test palbociclib plus cetuximab versus cetuximab alone, with OS as the primary endpoint [46].

### 4.2. Targeting PI3K/AKT/mTOR with Bimiralisib and Duvelisib

The phosphatidylinositol 3-kinase (PI3K)/AKT/mammalian target of rapamycin (mTOR) pathway is one of the most frequently dysregulated signaling cascades in HNSCC, with evidence of activation in over 90% of tumors across both HPV-positive and HPV-negative subsets [31,47,48]. This pathway governs essential cellular processes such as proliferation, survival, metabolism, angiogenesis, and motility, and its hyperactivation arises through multiple oncogenic mechanisms [49,50,51,52,53,54]. A major driver is receptor tyrosine kinase (RTK) signaling, particularly via the epidermal growth factor receptor (EGFR), which is overexpressed in 80–90% of HNSCC cases and signals downstream through PI3K, AKT, and mTOR to fuel tumor growth [55,56,57,58]. Genetic alterations also play a critical role, with gain-of-function mutations or amplifications in PIK3CA—encoding the p110α subunit of PI3K—present in roughly 30% of HNSCC lesions [59]. In addition, loss of the tumor suppressor PTEN, which normally antagonizes PI3K signaling, further enhances PI3K/AKT/mTOR activity [60].

Beyond tumorigenesis, the constitutive action of this pathway is a well-established mechanism of therapeutic resistance [61]. It undermines the efficacy of EGFR inhibitors such as cetuximab when accompanied by PTEN loss of P13K mutations, contributes to diminished benefit from CDK4/6 inhibitors, and promotes immune evasion, thereby reducing responsiveness to checkpoint blockade [61,62]. PI3K hyperactivation also fosters an immunosuppressive tumor microenvironment, although preclinical studies suggest that inhibition of this axis may resensitize tumors to PD-1 blockade by alleviating tumor hypoxia [63]. Given its central role in disease biology and treatment resistance, targeting the PI3K/AKT/mTOR pathway represents a promising therapeutic strategy [31,47,48]. Agents such as bimiralisib, a dual pan-PI3K/mTOR inhibitor, and duvelisib, a selective PI3Kδ/γ inhibitor, take this approach by aiming to disrupt proliferative signaling and overcome resistance mechanisms [64,65].

Bimiralisib (PQR309) is an oral, dual-acting inhibitor that selectively targets all four isoforms of Class I PI3K (α, β, γ, δ), as well as mTOR [66]. It has demonstrated activity in biomarker-selected subsets of R/M HNSCC [67]. Preclinical studies showed that bimiralisib suppresses proliferation and induces apoptosis in NOTCH1-mutant HNSCC models with dual PI3K/mTOR inhibition, proving more effective than targeting either pathway alone in HNSCC [67]. Importantly, HNSCC cells harboring loss-of-function (LOF) NOTCH1 mutations were more sensitive to PI3K/mTOR inhibition than wild-type counterparts, providing a strong rationale for its clinical development in genetically defined populations [67]. In a phase I study across advanced solid tumors, including HNSCC, bimiralisib displayed a manageable safety profile [68]. The maximum tolerated continuous daily dose was 80 mg, with grade 3 fatigue as the dose-limiting toxicity (DLT) [68]. Intermittent dosing (140 mg daily on two consecutive days weekly) was better tolerated, reducing the incidence of grade ≥ 3 adverse events, especially hyperglycemia (12% vs. 28.6% on continuous dosing) [68]. This intermittent schedule was chosen for further evaluation [68]. The most frequent treatment emergent adverse events were hyperglycemia (76.2% with continuous dosing) and nausea (56–62.5% with intermittent dosing) [68]. A prospective phase II study (NCT03740100) specifically enrolled patients with R/M NOTCH1-mutant HNSCC who had progressed after chemotherapy and immunotherapy [67]. Among eight patients treated (six evaluable), the study reported an ORR of 17%, including one patient with a confirmed partial response (49% reduction in target lesion size) [67]. mPFS was 5 months and OS was 7 months, outcomes that compared favorably to historical benchmarks for this population (ORR 5.8%, median OS 5.1 months, and PFS 2.7 months) [67]. Moreover, NOTCH1 mutations were detectable in circulating tumor DNA (ctDNA) in 83.3% of patients, supporting the feasibility of biomarker-based patient selection [67]. Although the trial was closed early due to sponsor insolvency, the data on bimiralisib in the biomarker-enriched population remains compelling for future development [67].

Compared with bimiralisib’s biomarker-driven strategy, duvelisib represents a complementary approach that leverages immune modulation and chemotherapy synergy to address resistance in broader, heavily pretreated HNSCC populations [64]. Duvelisib is an oral, dual, and selective PI3Kδ/γ inhibitor designed to target isoforms predominantly expressed in leukocytes, thereby modulating both innate and adaptive immune function [64]. Through this mechanism, duvelisib exerts an immunomodulatory effect that has been hypothesized to reverse resistance to ICIs and enhance tumor sensitivity to chemotherapy [64]. This rationale provided the basis for a phase II single-arm trial (NCT05057247) that evaluated duvelisib in combination with docetaxel in patients with anti-PD-1 refractory R/M HNSCC [64]. The combination met its primary endpoint, achieving an ORR of 19% (5 of 26 patients, all partial responses), with an additional stable disease rate of 46% (12 of 26 patients) [64]. Clinical outcomes in this heavily pretreated population were modest, with a mPFS of 2.8 months (95% Cl, 1.9–7.0) and a median OS of 10.2 months (95% Cl, 6.7–15.9) [64]. A trend toward longer OS was observed in HPV-negative patients [64]. The safety profile, however, was significant: 50% of patients (13 of 26) experienced grade ≥ 3 TRAEs and 25% (6 of 24) discontinued therapy due to toxicity [64]. Elevated liver function tests were the most common TRAE (23%), frequently leading to discontinuation of one or both agents, though no treatment-related deaths were reported [64]. Exploratory analyses further indicated that greater tumor infiltration by CD3+/CD8+ T cells correlated with improved outcomes, consistent with the proposed immunomodulatory mechanism [64]. Collectively, these findings highlight that duvelisib combined with docetaxel can synergize to overcome resistance in anti-PD-1 refractory HNSCC, though its therapeutic window may be limited by toxicity [64].

### 4.3. Targeting RAS/MAPK with Tipifarnib

The RAS/MAPK signaling cascade (RAS-RAF-MEK-ERK) is a critical driver of tumor cell growth, survival, and differentiation in HNSCC [55]. Acting downstream of EGFR and other RTKs, this pathway plays a central role in tumor progression [69]. Among the three PAS isoforms, HRAS is the most commonly mutated in HNSCC, with activating alterations occurring in approximately 4–8% of cases [70]. Clinically, these mutations define a small but important subset of R/M HNSCC patients, typically associated with poor prognosis and resistance to standard therapies [71]. Unlike KRAS or NRAS, which can bypass blockade of farnesyl transferase (FTase) through alternative prenylation (geranylgeranylation), HRAS is uniquely dependent on farnesylation to anchor to the cell membrane and activate downstream signaling [72]. This vulnerability provides a therapeutic entry point: tipifarnib, a selective FTase inhibitor, prevents farnesylation of HRAS at its C-terminal CAAX motif, trapping the protein in the cytosol and rendering it inactive [73]. Preclinical studies demonstrated that tipifarnib disrupts HRAS membrane localization and signaling, leading to tumor regression in HRAS-mutant HNSCC models, with efficacy tightly dependent on HRAS mutation status [74]. Activity was further enhanced by combinations with PI3K or ERK pathway inhibitors, and tipifarnib was shown to upregulate PD-L1 expression, suggesting potential synergy with immune checkpoint blockade [74]. In early phase I studies, tipifarnib established a manageable dosing schedule (600–900 mg orally twice daily, days 1–7 and 15–21 of a 28-day cycle), with hematologic toxicities such as anemia and lymphopenia as the most frequent adverse events, often requiring dose modifications [75]. Building on this foundation, the Phase II RUN-HN trial (NCT02383927) enrolled 22 patients with R/M HRAS-mutant HNSCC with high variant allele frequency (VAF ≥ 20%), of whom 20 were evaluable for response [75]. The trial reported an impressive ORR of 55% (95% Cl, 31.5–76.9), a mPFS of 5.6 months, and a median OS of 15.4 months in a heavily pretreated, immunotherapy-refractory population [75]. These outcomes compare favorably to the 5–15% response rates typically seen with standard later-line therapies in unselected HNSCC, leading to FDA breakthrough therapy designation and the launch of the AIM-HN trial (NCT03719690) [75]. The pivotal AIM-HN trial reported an ORR of 30% by investigator assessment and 20% by independent review, a solid response rate for a targeted therapy in a biomarker-selected HNSCC cohort [76,77]. The study remains ongoing to further evaluate efficacy and safety [76,77]. While tipifarnib administration and solid response rates in HRAS-mutant disease are clear advantages, its efficacy is limited to its molecularly defined subgroup making up 4–8% of HNSCC. Responses are often transient due to acquired resistance via PI3K/AKT activation, and hematologic toxicity can limit dosing [71,78]. Ongoing studies are therefore evaluating combinations such as PI3K inhibitors or immune checkpoint blockade (NCT04997902) [79].

### 4.4. Targeting IRS1/2 and the STAT3 Axis with NT219

The search for novel therapeutic strategies in HNSCC has increasingly focused on signaling pathways that drive resistance to established treatments, particularly the insulin receptor substrates (IRS1/2) and the signal transducer and activator of transcription 3 (STAT3) axis [80]. Dysregulation of these pathways plays a pivotal role in tumor progression and therapeutic resistance, especially to EGFR inhibitors [81]. Activated STAT3 is highly prevalent in HNSCC, detected in 37% to 75% of tumors, and is associated with an advanced disease stage and poor overall survival [80,82,83]. Similarly, upregulation of IGF1R/IRS and STAT3 signaling is frequently observed in HPV-negative R/M HNSCC, a population with particularly poor prognosis and limited treatment options [84,85]. Together, these data underscore the role of IRS1/2 and STAT3 in both tumor biology and therapy resistance, making them attractive targets for intervention [81].

Preclinical studies established a strong rationale for NT219 by identifying its unique mechanism as a small-molecule dual inhibitor that triggers degradation of IRS1/2 and suppresses STAT3 phosphorylation, two pathways known to mediate therapeutic resistance, particularly to EGFR inhibitors such as cetuximab [81]. In multiple patient-derived xenograft (PDX) models of HNSCC, NT219 combined with cetuximab produced a synergistic anti-tumor effect, with especially compelling activity in HPV-negative models [81]. Building on this rationale, a phase I/II clinical trial (NCT04474470) was initiated in immunotherapy-refractory R/M HNSCC [86]. The phase I dose-escalation portion employed a 3+3 design, enrolling 17 heavily pretreated patients across five NT219 dose levels (6–100 mg/kg), all of whom had previously failed anti-PD-1 therapy [86]. The combination of NT219 and cetuximab was found to be safe and well tolerated, with the most common TRAEs being infusion reactions, nausea, and rash [86]. Grade 3 events, including hypertension, infusion reaction, and headache, were infrequent, and no grade 4 or 5 events were reported [86]. Preliminary phase II results from the higher-dose cohorts (50 and 100 mg/kg NT219) demonstrated early but promising anti-tumor activity [86]. Among six evaluable patients treated at these target doses, the combination achieved an ORR of 33% (including two confirmed partial responses) and a DCR of 67% [86]. All patients who achieved disease control were HPV-negative and had previously progressed after immunotherapy containing regimens, highlighting a potential niche for this strategy in a particularly high-risk subgroup [86]. Exploratory biomarker analysis suggested that elevated activated IGF1R and STAT3 in pretreatment biopsies correlated with response, raising the possibility of biomarker-driven patient selection in future studies [81]. While these findings are encouraging, they remain preliminary given the small sample size, and further investigation in larger cohorts will be required to validate NT219’s clinical utility.

### 4.5. Targeting HER with Afatinib

The challenge addressed by the NT210 plus cetuximab combination—overcoming acquired resistance to EGFR blockade—reflects a central theme in HNSCC research, as EGFR is overexpressed in 80% to 100% of cases [87]. While cetuximab provides initial clinical benefit, its efficacy is consistently undermined by inherent and acquired resistance mechanisms, which involve activation of compensatory RTK pathways, most notably HER2, HER3, MET, and IGF-1R [88,89]. HER2 and HER3 are frequently overexpressed in cetuximab-resistant HNSCC, suggesting that dual or pan-HER inhibition may be required to achieve more durable responses [90]. This rationale underpins the investigation of afatinib, an irreversible pan-HER inhibitor either alone or in combination with cetuximab, as a strategy to provide comprehensive blockade across the entire ErbB receptor family (EGFR, HER2, HER3, and HER4) [91].

The rationale for combining afatinib with cetuximab in R/M HNSCC is based on preclinical studies showing that resistance to EGFR inhibition frequently arises through compensatory signaling mediated by other ErbB family members, particularly HER2 and HER3 [92]. Afatinib targets the intracellular ATP-binding domain of all ErBb family members, while cetuximab blocks the extracellular domain of EGFR [17,93]. Together, this dual blockade provides “vertical EGFR inhibition,” designed to overcome resistance by disrupting EGFR/HER dimers and suppressing non-canonical EGFR activities, such as nuclear translocation [94]. In a Phase 1b study of advanced solid tumors, including HNSCC, the combination established a maximum tolerated dose (MTD) and RP2D of afatinib 40 mg orally daily with cetuximab 250 mg/m^2^ weekly [91]. The regimen was reported to be generally tolerable, with diarrhea (63.8%) and acneiform dermatitis (43.1%) as the most common TRAEs [91]. The trial observed a confirmed DCR of 53.4% overall, reaching 66.7% in the HNSCC cohort, with a mPFS of 2.6 months overall [91]. Building on these findings, a Phase II trial enrolled 50 patients with R/M HNSCC refractory to platinum-based chemotherapy and/or immune checkpoint therapy; 47 patients were evaluable for response [93]. The combination achieved an ORR of 23.4%, with a mPFS of 3.8 months and a median OS of 7.5 months [93]. Importantly, efficacy was strongly influenced by HPV status, with an ORR of 38.5% in HPV-negative patients compared to only 4.8% in HPV-positive patients [93]. Although afatinib monotherapy previously failed to demonstrate an OS benefit over methotrexate in the second-line setting, the combination with cetuximab offers a clinically meaningful response rate in HPV-negative treatment-refractory HNSCC [95].

## 5. Targeted Therapies: Bispecific Antibody-Based Therapies 

The challenge highlighted by strategies such as afatinib plus cetuximab is the remarkable ability of HNSCC cells to evade treatment through compensatory signaling, including upregulation of HER2/HER3 or alternative RTKs such as MET and IGF-1R [88,89]. To move beyond the limitations of co-administering multiple agents, therapeutic development has advanced toward next-generation biologics capable of addressing multiple resistance patterns simultaneously [96]. Bispecific antibodies (BsAbs) are engineered molecules designed to target two distinct epitopes at once [97]. By doing so, BsAbs can achieve dual pathway inhibition—blocking complementary survival signals on the same cancer cell—and can also engage the immune system directly by bridging tumor-associated antigens such as EGFR with immune effector receptors like CD3 on T cells or NKG2D on NK cells, thereby initiating targeted cytotoxicity [97]. This dual functionality holds considerable promise for overcoming acquired resistance and broadening therapeutic benefit in R/M HNSCC. Emerging BsAbs, such as petosemtamab (EGFRxLGR5), are now leveraging this approach in clinical trials to improve outcomes in this population [98].

### 5.1. Petosemtamab (MCLA-158) Monotherapy

Petosemtamab (MCLA-158) is a human, common light-chain IgG1 bispecific antibody that simultaneously targets the EGFR and the leucine-rich repeat-containing G-protein-coupled receptor 5 (LGR5), a cancer stem cell marker [99]. Its antitumor activity is driven by a threefold mechanism of action [100]. First, it directly inhibits EGFR signaling by binding to domain III of the receptor and blocking ligand interaction [100]. Second, by simultaneously engaging both LGR5 and EGFR, the antibody facilitates rapid endocytosis and degradation of EGFR in LGR5-positive tumor cells—a mechanism not observed in cetuximab [100]. Third, petosemtamab is engineered with an afucosylated Fc region that enhances binding to activating Fc receptors, thereby maximizing antibody-dependent cellular cytotoxicity (ADCC) and antibody-dependent cellular phagocytosis (ADCP) against EGFR- and LGR5-expressing cells [100]. The preclinical rationale for petosemtamab is supported by evidence that LGR5 is upregulated in a wide range of solid tumors, including up to 89% of HNSCC cases, and is often induced following EGFR inhibition [98]. Studies in patient-derived organoid and xenograft models showed that petosemtamab more effectively inhibited tumor growth and metastasis than cetuximab, highlighting its unique capacity to exploit LGR5 upregulation as a therapeutic vulnerability [100]. 

In Phase I dose-escalation studies, the RP2D was established at 1500 mg intravenously every two weeks [101]. The overall safety profile was favorable, with relatively low rates of skin and gastrointestinal toxicity [100]. The most notable adverse events were infusion-related reactions (IRRs), which occurred in up to 66.7% of patients (22.2% grade ≥ 3), most commonly during the first infusion; these events were generally manageable and resolved with supportive care [100]. The subsequent phase II trial (NCT03526835) evaluated petosemtamab monotherapy in previously treated R/M HNSCC patients who had progressed on platinum-based chemotherapy and anti-PD-1/L1 therapy [101]. Among 75 evaluable patients, confirmed ORRs ranged from 36% to 40.4% [101]. Survival outcomes included an mPFS of 5.0–5.1. months and a median OS of 11.5–12.5 months, substantially exceeding historical benchmarks for second-line therapy, where ORRs are typically 6–19% and median OS 5.3–8.9 months [13,101]. These encouraging results supported advancement into the ongoing registration-intent phase III LiGeR-HN2 trial (NCT06496178) [102]. This randomized, open-label study is enrolling approximately 500 patients with R/M HNSCC whose disease has progressed after both anti-PD-1 and platinum-based treatment [102]. Petosemtamab monotherapy is being compared to the investigator’s choice of cetuximab, methotrexate, or docetaxel, with the goal of confirming its efficacy and safety [103].

### 5.2. Ficlatuzumab (HGF) + Cetuximab

The promising results observed with petosemtamab underscore the therapeutic potential of overcoming single-agent EGFR resistance by simultaneously targeting both survival and stemness pathways. One of the major mechanisms underlying acquired resistance to EGFR inhibition is compensatory signaling through alternate RTKs, particularly c-MET [104]. To address this bypass mechanism, investigators have pursued dual-targeting strategies that combine EGFR blockade with inhibition of c-MET-driven signaling [105]. This rationale led to the clinical development of ficlatuzumab, an anti-hepatocyte growth factor (HGF) monoclonal antibody that sequesters the HGF ligand and thereby disrupts c-MET activation, evaluated in combination with cetuximab [106].

Ficlatuzumab is a humanized IgG1 monoclonal antibody that binds with high affinity to HGF, the ligand for the c-MET RTK [106]. c-MET signaling is frequently dysregulated and highly activated in HPV-negative HNSCC, where it drives invasion, metastasis, and epithelial-to-mesenchymal transition (EMT), a phenotype associated with resistance to cetuximab [104,107,108]. By sequestering HGF, ficlatuzumab prevents c-MET activation, disrupting this major compensatory resistance mechanism to EGFR blockade [106]. Combining ficlatuzumab with cetuximab thus represents a rational dual-pathway inhibition strategy [109]. Preclinical studies supported this rationale, demonstrating that ficlatuzumab significantly reduced tumor-associated fibroblast-facilitated proliferation, migration, and invasion in HNSCC models by mitigating HGF/c-MET signaling and downstream MAPK phosphorylation [106].

The phase I dose-escalation study established the RP2D as ficlatuzumab 20 mg/kg plus cetuximab 500 mg/m^2^ every two weeks [110]. The regimen was tolerable, with no dose-limiting toxicities observed, and showed preliminary efficacy in a heavily pretreated, cetuximab-resistant HNSCC population, achieving a confirmed ORR of 17% and a mPFS of 5.4 months [110]. These findings led to a randomized phase II trial (NCT03422536) evaluating ficlatuzumab with or without cetuximab in pan-refractory R/M HNSCC resistant to platinum, anti-PD-1 therapy, and cetuximab [109]. The combination arm of 32 patients achieved an mPFS of 3.7 months, meeting prespecified criteria for further development [109]. Notably, the benefit was highly stratified by HPV status: the HPV-negative subgroup (*n* = 16) demonstrated substantial activity with an ORR of 38% (including 2 complete and 4 partial responses), mPFS of 4.1 months, and median OS of 7.4 months [109]. In contrast, the HPV-positive cohort derived minimal benefit, with an ORR of 0% and mPFS of only 2.3 months [109]. Based on these encouraging signals in HPV-negative disease, the combination advanced to the ongoing phase III FIERCE-HN trial (NCT06064877) [111]. This randomized, double-blind, placebo-controlled study is enrolling approximately 410 patients with HPV-negative R/M HNSCC who have progressed on anti-PD-1/L1 and platinum-based chemotherapy [111]. Its primary endpoint is overall survival, comparing ficlatuzumab plus cetuximab versus placebo plus cetuximab [111].

## 6. Targeted Therapies: Antibody-Drug Conjugates (ADCs)

Another rapidly advancing class of next-generation biologics is antibody-drug conjugates (ADCs). Unlike BsAbs, ADCs represent a distinct mechanistic strategy: rather than relying solely on antibody-mediated immune effects such as ADCC or pathway inhibition, ADCs combine the precision targeting of a monoclonal antibody with the localized delivery of a cytotoxic chemotherapy payload [112]. This design enhances antitumor efficacy while reducing the systemic toxicity often associated with conventional chemotherapy [113]. Several ADCs targeting antigens highly expressed in HNSCC are currently demonstrating promising activity in clinical trials within the recurrent and metastatic setting.

### 6.1. MRG003 (Becotatug Vedotin)—EGFR-Targeted ADC

MRG003 is an antibody–drug conjugate (ADC) composed of a humanized anti-EGFR IgG1 antibody linked to a monomethyl auristatin E (MMAE) payload via a cleavable valine–citrulline linker [114]. This design enables selective binding to EGFR-expressing tumor cells, internalization, and intracellular release of the cytotoxic agent, resulting in mitotic arrest and cell death [115]. The cleavable linker also confers a bystander effect, allowing payload diffusion to neighboring tumor cells with lower EGFR expression and thereby broadening antitumor activity [114]. The drug-to-antibody ratio (DAR) of approximately 3.8 optimizes the balance between efficacy and tolerability [114]. Preclinical studies demonstrated potent antitumor activity in EGFR-expressing xenograft models through the selective delivery of MMAE, resulting in targeted tumor cell death while potentially minimizing systemic toxicity [114]. In a multicenter Phase I trial with a Phase Ib expansion, MRG003 was administered at a recommended dose of 2.5 mg/kg every three weeks in patients with refractory, EGFR-positive advanced HNSCC [114]. Among 13 patients assessed, the ORR was 40%, and the DCR reached 100% with manageable toxicities [114]. Most adverse events were grade 1–2, while grade ≥ 3 events (31%) included hyponatremia, leukocytopenia, neutropenia, and elevated liver enzymes [114]. Building on these findings, a Phase 2 study in heavily pretreated EGFR-positive HNSCC patients (62 total) reported a confirmed ORR of 43% and a DCR of 86% at the 2.3 mg/kg dose level, with a mPFS of 4.2 months and a median overall survival (OS) of 11.3 months [115]. A Phase 3 trial (NCT05751512) is currently underway, comparing MRG003 to the investigator’s choice of cetuximab or methotrexate in recurrent or metastatic HNSCC patients who have progressed on anti–PD-1 and platinum-based chemotherapy [116].

### 6.2. Tisotumab Vedotin (TV)—Tissue Factor (TF)-Targeted ADC

The success of EGFR-targeted ADCs such as MRG003 validates the therapeutic strategy of exploiting highly expressed surface proteins to deliver potent cytotoxic payloads directly to tumor cells. This principle has been extended to other tumor-associated antigens, generating significant interest in tissue factor (TF)–targeted ADCs [117,118]. TF, also known as coagulation factor III or CD142, is a 47 kDa transmembrane glycoprotein that normally resides in subendothelial tissues, where it serves as the primary initiator of the extrinsic coagulation cascade following vascular injury [119,120]. In cancer, however, TF is frequently overexpressed across a wide range of solid tumors, including HNSCC, where elevated TF levels are associated with poor prognosis, enhanced metastasis, and angiogenesis [121,122]. These features make TF an attractive, though biologically complex, target for ADC development. The greatest challenge lies in mitigating bleeding-related toxicities given TF’s central role in clotting [123]. To address this, next-generation anti-TF ADCs are being engineered with refined linker and payload technologies to enhance tumor specificity while minimizing systemic toxicity. Tisotumab vedotin (TV) is a first-in-class ADC that targets TF, which is variably expressed in HNSCC and associated with poor prognosis [121]. TV consists of a TF-directed monoclonal antibody linked to the cytotoxic payload MMAE via a cleavable linker, allowing for targeted internalization and subsequent microtubule disruption that drives tumor cell death [124]. Preclinical studies demonstrated potent antitumor activity in solid tumor models, supporting its advancement into clinical testing [125]. TV is FDA-approved for the treatment of R/M cervical cancer in adults whose disease has progressed on or after chemotherapy [126]. The Phase 2 innovaTV 207 trial (NCT03485209) enrolled patients with recurrent or metastatic HNSCC who had progressed on prior platinum chemotherapy and immune checkpoint inhibitors [127]. In this heavily pretreated population of 40 patients, TV achieved an ORR of 32.5% with a median duration of response (DOR) of 5.6 months, with even higher efficacy (ORR 40%) observed in patients treated in the second- or third-line setting [128]. Notable toxicities were observed, with 40% of patients experiencing bleeding of any grade, 47.5% peripheral neuropathy, and 52.5% ocular toxicities [128]. Keeping in mind the toxicity profile, these results compare favorably to cetuximab monotherapy, which historically achieves an ORR of only 10–13% in similar populations [13,14,15]. This ongoing study is actively investigating whether efficacy can be further enhanced, with the goal of defining TV’s role in HNSCC [128].

### 6.3. Enfortumab Vedotin (EV)—Nectin-4-Targeted ADCs

Another ADC target is Nectin-4, a cell-adhesion molecule that is highly expressed in the majority of head and neck cancers, with studies reporting expression in up to 86.2% of HNSCC cases [129]. This high prevalence makes Nectin-4 an attractive therapeutic target. Enfortumab vedotin (EV) is a Nectin-4-directed ADC that delivers the cytotoxic payload MMAE [130]. Structurally, EV consists of an anti–Nectin-4 IgG1 antibody conjugated to MMAE via a protease-cleavable linker [130]. Upon binding to Nectin-4–expressing tumor cells, EV undergoes internalization, and the MMAE payload is released intracellularly [130]. Preclinical studies demonstrated not only potent antitumor activity in Nectin-4–expressing models but also induction of immunogenic cell death, thereby activating immune responses and providing a rationale for evaluating EV in combination with immune checkpoint inhibitors [130,131]. The first-in-human Phase I EV-101 trial established the safety and preliminary efficacy of EV across multiple Nectin-4–positive solid tumors with a manageable toxicity profile characterized by common treatment-related adverse events such as alopecia (28.3%), fatigue (26.1%), and peripheral sensory neuropathy (23.9%) [132]. The Phase II EV-202 trial (NCT04225117) assessed EV monotherapy in 46 heavily pretreated patients with R/M head and neck cancer who had progressed after platinum-based therapy and PD-1/PD-L1 inhibitors [133]. In this population, EV achieved a confirmed ORR of 23.9%, a DCR of 56.5%, and a median duration of response (DOR) of 9.4 months, with a mOS of 6.0 months [133]. Importantly, patients also reported improvements in cancer-related pain beginning in cycle 3 and beyond, underscoring quality-of-life benefits in addition to tumor shrinkage [134]. With these results, EV demonstrates meaningful clinical activity in heavily pretreated HNC, meriting further development [133].

### 6.4. Sacituzumab Govitecan (SG)—Trop-2-Targeted ADCs

Another highly prevalent target in HNSCC is Trophoblast Cell-Surface Antigen 2 (Trop-2). Trop-2 is widely expressed in HNSCC, and its overexpression correlates with adverse clinical outcomes, making it an attractive candidate for targeted therapy in heavily pretreated patients with advanced disease [135]. Sacituzumab govitecan (SG), the most advanced Trop-2–directed ADC, is engineered to deliver cytotoxic therapy specifically to Trop-2–expressing tumors, which include HNSCC [135,136]. It consists of a humanized anti-Trop-2 monoclonal antibody conjugated through a hydrolyzable linker to SN-38, the active metabolite of irinotecan and a potent topoisomerase I inhibitor [136,137]. Once bound to Trop-2 on the tumor-cell surface, SG is internalized and releases SN-38 intracellularly, inducing DNA damage and cell death [136,137]. The same cleavable linker permits a bystander effect, allowing SN-38 to diffuse into adjacent tumor cells with low or heterogeneous Trop-2 expression and thereby amplifying intratumoral cytotoxicity [136,138]. Preclinical work confirmed Trop-2 as a relevant therapeutic target across solid tumors and provided the rationale for clinical testing of SG [139]. In the early phase I/II basket study IMMU-132-01 (NCT01631552), SG produced preliminary antitumor activity in multiple epithelial cancers, including HNSCC, and established the recommended schedule of 10 mg/kg intravenously on days 1 and 8 of each 21-day cycle [140]. The pooled safety analysis from this program showed a manageable profile, with neutropenia (57.8%), diarrhea (56.2%), and nausea (62.6%) as the most common treatment-related adverse events [140]. The most compelling HNSCC-specific evidence derives from the phase II TROPiCS-03 trial (NCT03964727), which enrolled 43 heavily pretreated patients who had progressed after platinum chemotherapy and anti–PD-(L)1 therapy [141]. In this cohort, SG achieved an ORR of 16% (95% CI, 7–31%), a clinical-benefit rate of 28% (95% CI, 15–44%), and a median duration of response of 4.2 months (95% CI, 2.6–not reached) [141]. mPFS was 4.1 months (95% CI, 2.6–5.8) and mOS was 9.0 months (95% CI, 7.1–10.5) [141]. Grade ≥3 treatment-emergent adverse events occurred in 58% of patients—most commonly diarrhea (47%), nausea (47%), and neutropenia (47%)—but led to treatment discontinuation in only 2% of cases [141]. A phase II study (NCT07063212) of SG with cetuximab is currently recruiting patients with recurrent/metastatic HNSCC that has progressed after first-line therapy [142].

### 6.5. Sigvotatug Vedotin (SGN-B6A)—Integrin Beta-6 (ITGB6)-Targeted ADCs

Additional tumor-associated receptors in HNSCC include integrin beta-6 (ITGB6), a component implicated in tumor invasiveness and frequently overexpressed in HNSCC, where its expression is associated with poor clinical outcomes [143]. To exploit this vulnerability, sigvotatug vedotin (SV), formerly known as SGN-B6A, has been developed as an ITGB6-directed ADC, representing a novel approach to targeted therapy in HNSCC and other ITGB6-driven malignancies [143]. The sigvotatug vedotin construct links an ITGB6-directed monoclonal antibody to the cytotoxic payload MMAE via a cleavable linker, enabling selective internalization and microtubule disruption in ITGB6-expressing tumor cells [143]. Preclinical studies demonstrated potent antitumor activity in pharyngeal carcinoma xenograft models, with selective cytotoxicity and no significant off-target toxicity at clinically relevant doses, supporting clinical development [143]. In the Phase I SGNB6A-001 trial (NCT04389632), SGN-B6A monotherapy in 56 heavily pretreated HNSCC patients achieved an ORR of 20–23% and a DCR of 61% [144]. However, grade ≥ 3 adverse events occurred in 44.5% of patients, most commonly fatigue, neutropenia, and peripheral sensory neuropathy, though toxicities were generally manageable [144]. These findings support further investigation, with Phase II dose-expansion cohorts in HNSCC actively enrolling patients with recurrent or metastatic disease post-immunotherapy [144].

### 6.6. Ozuriftamab Vedotin (BA3021)—ROR2-Targeted ADCs

Attention has turned to Receptor Tyrosine Kinase-like Orphan Receptor 2 (ROR2). ROR2 is a transmembrane receptor tyrosine kinase that is up-regulated by the HPV-E6/E7 oncoproteins and is overexpressed in several solid tumors, including HNSCC, where high expression correlates with aggressive behavior and treatment resistance [145]. Ozuriftamab vedotin (BA3021) is the first-in-class conditionally active biologic (CAB) ADC designed to exploit this vulnerability by selectively targeting ROR2-positive malignancies [146]. Ozuriftamab vedotin’s CAB design allows it to selectively and reversibly bind ROR2 under the acidic conditions of the tumor microenvironment, minimizing off-target binding in normal tissues [146]. Once bound and internalized, the protease-cleavable vedotin linker releases the cytotoxic payload MMAE [146]. Pre-clinical studies showed potent, selective lysis of ROR2-expressing cell lines and durable tumor regression in xenograft models, while non-human primates tolerated single doses up to 10 mg/kg with good systemic stability [146]. In the first-in-human phase I study (NCT03504488) of advanced solid tumors—including HNSCC—the RP2D was 1.8 mg/kg, neuropathy and neutropenia were the main dose-modifying toxicities, and durable objective responses were recorded [147,148]. In the ongoing Phase II trial (NCT05271604), 31 heavily pretreated patients with recurrent or metastatic HNSCC (median of three prior lines) who had progressed on prior PD-1 therapy were enrolled, with efficacy assessed in 25 evaluable patients [149]. BA3021 achieved an ORR of 32% (8/25), including one complete response, and stable disease in 40% of patients, while in the HPV-associated HNSCC subset (*n* = 11), the mPFS was 4.8 months and median OS was 11.6 months [149]. Grade 3–4 events have been infrequent, limited mainly to anemia, hyponatremia, and hypoxia, while the most common treatment-related events are fatigue (55%) and nausea (29%) [149]. Based on these encouraging results in an immunotherapy-refractory population, BA3021 received U.S. FDA Fast Track Designation for recurrent/metastatic HNSCC in July 2024, supporting further clinical development, including evaluation in earlier lines of therapy. An upcoming phase III trial will be crucial to determine if this drug moves forward. If phase III results are promising, BA3021 will likely become a new standard of care.

## 7. Next-Generation Therapies: Vaccines

Beyond conventional targeted agents and standard checkpoint inhibitors, a new wave of therapeutic strategies is being explored for R/M HNSCC [150]. These novel approaches aim to overcome both primary and acquired resistance by directly modulating the immunosuppressive TME or by exploiting highly specific tumor-associated antigens [151]. Innovative biological platforms such as therapeutic vaccines are being developed to stimulate or enhance the patient’s antitumor immune response, offering particular promise in the immunotherapy-refractory setting [152]. Therapeutic cancer vaccines aim to induce tumor regression, eliminate microscopic disease, and establish durable anti-tumor immune memory, and they are broadly classified by delivery mechanism (peptide, RNA, DNA, or tumor cell-based) and by target, typically directed against tumor-associated antigens (TAAs) or tumor-specific neoantigens (TSAs) [153].

### 7.1. MVX-ONCO-1

MVX-ONCO-1 is a personalized, cell-based vaccine designed to stimulate broad antitumor immunity by combining a patient’s irradiated autologous tumor cells—which provide the complete repertoire of individual tumor antigens—with encapsulated allogeneic cells genetically engineered to secrete granulocyte-macrophage colony-stimulating factor (GM-CSF) directly at the vaccination site [154]. This dual mechanism promotes antigen presentation and local immune activation, addressing the limitations of traditional cancer vaccines that often fail due to inefficient priming and weak adjuvants [154]. The encapsulated GM-CSF delivery system allows for sustained, localized cytokine release over several days without systemic exposure [154]. In early Phase I studies (NCT02999646) across advanced solid tumors, including R/M HNSCC, MVX-ONCO-1 demonstrated safety and feasibility, with no systemic adverse events attributed to the vaccine and signals of clinical benefit, including partial responses and prolonged survival [155]. Remarkably, one R/M HNSCC patient who had progressed after chemotherapy and anti–PD-1 therapy survived more than seven years, remaining free of anticancer treatment for over five years [155]. The most compelling evidence comes from the Phase II SAKK 11/16 trial, which enrolled heavily pretreated R/M HNSCC patients, 85% of whom had previously progressed on anti–PD-1 therapy [154]. In this difficult-to-treat population, MVX-ONCO-1 achieved its primary endpoint, with 68.8% of patients alive at six months and a median overall survival (OS) of 11.4 months (95% CI, 4.4–NR) [154]. The 18-month OS rate was 31.6%, outcomes that compare favorably to historical benchmarks [154]. While the objective response rate (ORR) was modest, including both complete and partial responses, clinical benefit correlated strongly with the induction of an immune response [154]. All patients who developed a positive delayed-type hypersensitivity (DTH) reaction to their tumor cells survived at 12 months, highlighting its potential as a predictive biomarker [154]. Treatment was well tolerated, with only mild to moderate local adverse events and no new systemic safety concerns [154]. These findings support further clinical development of MVX-ONCO-1, particularly in combination with immune checkpoint inhibitors, where prior ICI exposure may enhance its therapeutic effect [154].

### 7.2. ISA101 (Peltopepimut-S)

The field of therapeutic vaccines includes multiple platforms aimed at overcoming resistance, with notable success seen in targeting HPV-positive tumors [150]. In this subset of cancers, the viral oncoproteins E6 and E7 are consistently expressed and essential for malignant cell survival, making them ideal, well-defined targets [150]. Capitalizing on this biology, ISA101 (peltopepimut-S), a synthetic long-peptide vaccine encompassing HPV16 E6 and E7 antigens, was developed to specifically expand CD4^+^ helper and CD8^+^ cytotoxic T-cell responses against these viral proteins, thereby generating a highly targeted antitumor immune response [156]. Preclinical studies demonstrated potent HPV16-specific T-cell activation and antitumor activity in animal models, providing the rationale for clinical development in HPV-driven malignancies such as HNSCC [157]. Early clinical results were promising: in a single-arm Phase II trial (NCT02426892), ISA101 combined with nivolumab achieved an ORR of 33% in advanced HPV16-positive cancers, rising to 36% in oropharyngeal cancer, with a median OS of 17.5 months, leading the FDA to grant Fast Track designation to ISA101b for recurrent/metastatic (R/M) HPV16-positive HNSCC [158,159]. In the post–PD-1 refractory setting, a Phase II trial (NCT04398524) of ISA101b plus cemiplimab in HPV16-positive oropharyngeal cancer reported modest response rates, with an ORR of 6.3% (11.5% in stage 1) but a higher clinical benefit rate (CBR) of 56.3% (61.5% in stage 1) and a median OS of 11.3 months [160]. Importantly, some patients who had not responded to prior PD-1 therapy achieved partial responses, suggesting ISA101b may help overcome resistance in select subsets [161]. The regimen was generally well tolerated, with grade 3 adverse events such as erythema, diarrhea, and immune-related toxicities occurring in a minority of patients (4.7–7.7%) and no grade 4–5 events [160]. Ongoing research continues to assess this platform in combination with immune checkpoint inhibitors.

### 7.3. CUE-101

The clinical outcomes observed with ISA101 reinforce the therapeutic potential of stimulating antigen-specific T cells against HPV16 E7 in refractory HNSCC. A novel approach to achieving this same targeted stimulation is embodied by CUE-101, the lead compound from the Immuno-STAT (Selective Targeting and Alteration of T cells) platform [162]. Unlike peptide vaccines, this engineered fusion protein delivers both the antigen-specific T-cell receptor (TCR) engagement signal—via an HPV16 E7 peptide presented in the context of HLA-A02:01—and an affinity-attenuated interleukin-2 (IL-2) proliferation signal within a single molecule [162]. By integrating these components, CUE-101 ensures selective activation and expansion of only the desired HPV16 E7–E7-specific CD8^+^ T cells, representing a next-generation strategy for precise and durable T-cell immunotherapy [162]. Preclinical studies demonstrated that CUE-101 effectively expanded HPV16 E7-specific CD8^+^ T cells from human PBMCs and, in HLA-A2 transgenic mouse models, increased tumor-infiltrating T cells, improved survival, and induced durable immunologic memory [162]. A surrogate construct (mCUE-101) in murine TC-1 tumor models also showed enhanced efficacy when combined with PD-1 blockade, supporting its clinical development [162]. In the ongoing Phase I/II trial (NCT03978689) in patients with recurrent/metastatic HPV16-positive HNSCC refractory to platinum-based and checkpoint inhibitor therapy, dose escalation established 4 mg/kg IV every three weeks as the RP2D, with no maximum tolerated dose reached [163]. CUE-101 was generally well tolerated, with common adverse events including fatigue, anemia, infusion reactions, and chills, most grade 1–2 [164]. Pharmacodynamic analyses confirmed selective expansion of HPV16 E7-specific CD8^+^ T cells in treated patients [164]. Among 19 evaluable patients treated at the recommended dose, CUE-101 monotherapy achieved a 37% clinical benefit rate, including one partial response and six cases of durable stable disease (≥12 weeks) [164]. Notably, the median overall survival in this heavily pretreated cohort was 24.4 months—essentially more than doubling historical controls [164]. Upcoming studies plan to further evaluate the efficacy and safety of CUE-101 in larger patient populations.

## 8. Comparative Evidence Synthesis Across Classes

Across phase II datasets in the post-ICI setting, three broad patterns emerge when therapies are compared head-to-head on practical outcomes: tumor response, durability/survival, and breadth of eligibility. First, for rapid cytoreduction, optimized chemotherapy backbones, especially paclitaxel + cetuximab, remain the ORR benchmark: prospective trials reported ORR 69.6% (jRCTs051200040) with mOS ~13.3 mo and ORR 47.4% (PACE-ACE) with mOS ~14.0 mo, albeit with expected grade ≥ 3 toxicities (e.g., neutropenia/infection) [7,18]. Second, biomarker-selected targeted strategies have delivered the strongest survival signals in defined subsets. For example, CDK4/6 inhibition + EGFR blockade (dalpiciclib + cetuximab) in HPV-negative, PD-1–resistant disease achieved ORR 67.9% and mOS 17.0 mo—clearly exceeding typical post-ICI historical controls—while maintaining a manageable safety profile [38,39]. Similarly, pathway-informed doublets such as ficlatuzumab (HGF) + cetuximab (EGFR) show preferential activity in HPV-negative tumors and have advanced to a Phase III OS-endpoint trial (FIERCE-HN) to test whether dual EGFR/HGF-c-MET blockade can outperform cetuximab-based control regimens [109,111]. Third, “platform” modalities with broad antigen coverage, notably ADCs and bispecific antibodies, are producing consistent activity across largely unselected, post-ICI cohorts and are now moving into registration-intent trials. Among ADCs, MRG003 (anti-EGFR-MMAE) has reported ORR 43% with mOS 11.3 mo in heavily pretreated EGFR-positive HNSCC and is in Phase III against investigator’s choice, underscoring class potency with acceptable tolerability for refractory patients [115,116]. Tisotumab vedotin (anti-TF-MMAE) achieved ORR 32.5% (40% in 2nd/3rd line) in innovaTV-207, surpassing historical cetuximab monotherapy benchmarks, and combinations with PD-1 blockade are under study [13,14,15,128]. Enfortumab vedotin (anti-Nectin-4-MMAE) demonstrated ORR 23.9%, DCR 56.5%, mDOR 9.4 mo and mOS 6.0 mo in a heavily pretreated HNC cohort, with noted pain improvements that add quality-of-life value and support combination exploration with PD-1 inhibitors [133,134]. Sacituzumab govitecan (anti-Trop-2-SN-38) showed ORR 16%, CBR 28%, mPFS 4.1 mo, mOS 9.0 mo in TROPiCS-03; despite higher rates of ≥G3 AEs typical of topoisomerase-I payloads, discontinuation was low (2%), supporting continued Phase III/combination development [141,142]. Ozuriftamab vedotin achieved an ORR 32%, mPFS 4.8 mo, and mOS 11.6 mo [149]. On the bispecific/novel immunotherapy front, petosemtamab (EGFR × LGR5) monotherapy has delivered ORR 36–40.4% with mOS 11.5–12.5 mo and is in a Phase III head-to-head against SOC options, illustrating the promise of dual-target biology for broadly eligible patients [13,101,102,103]. Meanwhile, CUE-101 (Immuno-STAT E7-pHLA-IL-2 fusion)—though restricted to HPV16+ and HLA-A02:01—has produced a notable median OS of 24.4 months with a 37% clinical-benefit rate as monotherapy, highlighting a durability signal in a narrow biomarker slice and pointing to synergy with PD-1 blockade [164].

### Qualitative Comparative Synthesis

Taken together, these findings highlight distinct strengths and limitations across therapeutic classes. Chemotherapies remain the most reliable option for immediate tumor shrinkage, critical in scenarios like airway compromise or bleeding risk, but their benefits are tempered by high toxicity and limited durability. Immunotherapies, particularly next-generation agents such as CUE-101 or vaccines like ISA101 and MVX-ONCO-1, show the capacity for durable disease control, yet their efficacy is restricted to biomarker-defined niches (e.g., HPV16+, HLA subtypes). Targeted small-molecule combinations (e.g., CDK4/6 + EGFR, tipifarnib in HRAS-mutant tumors) achieve survival outcomes that rival or exceed immunotherapies in selected subsets, but their applicability is constrained by the rarity of these molecular alterations. In contrast, antibody-based modalities, ADCs and bispecific antibodies, offer broad applicability across unselected or lightly selected populations. ADCs deliver potent cytotoxic payloads with consistent ORRs in the 20–40% range, though durability varies by payload class, while bispecifics provide dual-target engagement with mid-30% ORRs and favorable tolerability [13,14,15,101,102,103,115,116,128,133,134,149]. Finally, vaccine platforms occupy an intermediate space: they rarely induce rapid shrinkage, but they can prime durable antitumor immunity, particularly when paired with checkpoint inhibitors, suggesting a role in sustaining long-term disease control.

If immediate tumor shrinkage is the priority, paclitaxel + cetuximab remains the most reliable salvage comparator today, though toxicities and durability limits are present [7,18]. Where actionable biology is present (e.g., HPV-negative with cell-cycle activation; EGFR/HGF-c-MET crosstalk), targeted combinations can match cytoreduction and extend survival up to approximately 15 months, pending Phase III validation [38,39]. For broadly eligible, post-ICI populations, ADCs and bispecifics are emerging as practical alternatives that deliver mid-30% ORRs at the top end (petosemtamab; MRG003) with manageable, class-specific toxicities; several are already in registration-intent trials that could redefine the standard in unselected or lightly selected cohorts [13,101,102,103,115,116]. Finally, next-gen immunotherapies (e.g., CUE-101) offer durability in tightly defined niches, suggesting that the field is converging on biomarker-guided, mechanism-based combinations as the path to outperform chemotherapy comparators in Phase III [164]. Given the biological divergence between HPV-positive and HPV-negative head and neck squamous cell carcinoma, treatment efficacy and immune responsiveness often depend on viral status. To illustrate these distinctions, Table 1, Table 2 and Table 3 categorize current and emerging therapies by HPV association—HPV-negative, HPV-positive, and HPV-neutral—summarizing each agent’s mechanism of action, clinical development stage, and efficacy outcomes.

## 9. Challenges and Future Directions

A critical challenge in the post-ICI setting is the transition of promising novel agents from early-phase, single-arm trials to definitive, randomized Phase III studies that can establish a new standard of care. Many emerging therapies, including ADCs and targeted combinations, have demonstrated compelling ORRs in Phase II studies. However, the absence of robust control arms limits definitive conclusions about overall survival benefit and true superiority over established regimens. To meaningfully address the post-ICI gap, novel agents must demonstrate superior efficacy compared to the current active salvage chemotherapy benchmark, paclitaxel plus cetuximab (mOS 13.3–14.0 months, ORR up to 69.6%) [7,18]. Several registration-intent Phase III trials are underway, including LiGeR-HN2 (petosemtamab, EGFR × LGR5), FIERCE-HN (ficlatuzumab + cetuximab), and Phase III ADC studies such as MRG003, all of which compare novel regimens against the investigator’s choice of standard options [103,111,116].

Patients with R/M HNSCC after ICI failure are often heavily pretreated and frail, making tolerability a major barrier. Standard chemotherapy regimens such as paclitaxel plus cetuximab achieve high ORRs but carry grade ≥ 3 toxicity rates up to 65%, driven by neutropenia and infection [7,18]. Novel classes bring unique toxicity profiles: PI3K inhibition combined with chemotherapy results in frequent high-grade adverse events, while ADCs show payload-specific toxicities (e.g., neuropathy and cytopenias with vedotin-based agents; diarrhea and neutropenia with topoisomerase I inhibitor payloads like sacituzumab govitecan) [64,128,132]. Future development must focus on agents that balance efficacy with tolerability, such as nanoparticle formulations (e.g., FID-007) designed to improve drug delivery and reduce systemic toxicity [19]. 

Durable efficacy in the post-ICI setting is increasingly confined to biomarker-defined niches, necessitating the systematic use of next-generation sequencing (NGS), circulating tumor DNA (ctDNA), and molecular typing. Targeted small-molecule combinations demonstrate their strongest efficacy in defined subsets—for example, dalpiciclib plus cetuximab (mOS 17.0 months) in HPV-negative, PD-1–resistant tumors, or tipifarnib in HRAS-mutant disease [38,39,71,78]. Similarly, NOTCH1-mutant HNSCC shows sensitivity to PI3K/mTOR inhibition, and CDKN2A-altered tumors may benefit from CDK4/6 inhibition [45,67]. ctDNA is emerging as a key tool, both for detecting actionable alterations in real time (e.g., NOTCH1 mutations identified in 83.3% of patients) and for longitudinally tracking clonal evolution, enabling adaptive sequencing of therapies [67]. In parallel, molecular typing approaches such as mutational or “Cosmic” signatures (e.g., smoking-associated, aging-related) may allow tumors to be grouped into biologically coherent subtypes, guiding treatment toward pathway-specific inhibitors or immunotherapies. Together, ctDNA and mutational signatures hold promise for refining patient selection, reducing trial heterogeneity, and creating a foundation for adaptive trial designs that test therapies in increasingly granular subgroups.

Another major future direction involves reprogramming the immunosuppressive TME to restore PD-1 sensitivity. Bifunctional fusion proteins such as retlirafusp alfa (PD-L1 × TGF-β trap) aim to overcome T-cell exclusion, while CCR4 antagonists like FLX475 block Treg recruitment, thereby restoring cytotoxic T-cell activity [25,27]. Therapeutic vaccines represent complementary strategies for immune priming, aiming to convert immune “cold” tumors into “hot,” checkpoint-sensitive ones. Intriguingly, retrospective analyses suggest that prior ICI exposure may “prime” tumors, with subsequent chemotherapy achieving higher response rates than expected historically, highlighting the potential of re-sensitization strategies [6]. The field is converging on highly personalized, mechanism-driven approaches. Personalized vaccines such as MVX-ONCO-1 use autologous tumor cells to present a full antigen repertoire, while engineered fusion proteins such as CUE-101 demonstrate durability in HPV16+ HLA-A*02:01-restricted disease (mOS 24.4 months) [154,164]. Adaptive trial designs will be essential to efficiently evaluate such therapies in stratified populations defined by HPV status, PD-L1 expression, mutational alterations, and molecular signatures. The future of post-ICI HNSCC therapy lies in integrating biomarker-driven strategies, immunologic re-sensitization, and adaptive trial platforms to ultimately deliver durable survival beyond what chemotherapy comparators can achieve.

## 10. Conclusions

The challenge of R/M HNSCC after immune checkpoint inhibitor (ICI) failure represents one of the most pressing unmet needs in oncology. Up to 80% of patients experience progression following ICI therapy, and historical salvage options rarely extend survival beyond a year [2]. The current benchmark for highly active salvage remains paclitaxel plus cetuximab, with ORRs approaching 70% and median OS of 13–14 months, though this regimen carries significant grade ≥ 3 toxicities [7,18]. Closing this therapeutic gap requires novel agents that not only match chemotherapy in response rates but also provide superior durability and tolerability.

Comparative evidence across therapeutic classes highlights distinct advantages and limitations. Biomarker-driven targeted combinations, such as dalpiciclib plus cetuximab and tipifarnib, demonstrate the highest efficacy in defined molecular subsets—particularly HPV-negative, PD-1–resistant, and HRAS-mutant disease. These results rival or exceed chemotherapy, but their applicability is limited by the rarity of these alterations. In contrast, antibody-based platform modalities offer broader applicability across unselected patients. Petosemtamab (EGFR × LGR5 bispecific) exemplifies the promise of dual-targeted receptor blockade, while ADCs such as MRG003 (EGFR-directed) and tisotumab vedotin (TF-directed) combine targeted delivery with potent cytotoxic payloads to achieve consistent disease control. Although payload-specific toxicities remain a consideration, these agents are rapidly advancing into registration-intent Phase III trials. Finally, next-generation immunotherapies and vaccines like CUE-101 have demonstrated the capacity for durable disease control in biomarker-restricted niches, underscoring the potential of engineered immune therapies to extend survival in subsets where precision targeting is possible.

Taken together, these comparative insights show a field in transition from reliance on non-specific chemotherapy to the integration of mechanism-based, personalized approaches. To establish a definitive new standard of care, future development must focus on three pillars: (1) biomarker guidance, using next-generation sequencing (NGS), circulating tumor DNA (ctDNA), and molecular signatures to match patients to targeted therapies and guide sequencing; (2) toxicity management, ensuring that novel agents—such as nanoparticle formulations and conditionally active biologics—preserve efficacy while reducing systemic toxicity in heavily pretreated populations; and (3) adaptive trial designs, embedding biomarker selection into Phase III studies to efficiently test novel combinations against the established chemotherapy benchmark. Ultimately, the integration of these modalities, guided by biomarkers and tested in adaptive trial frameworks, holds the promise of redefining the treatment paradigm and delivering durable, tolerable survival benefits.

## Figures and Tables

**Table 1:** Emerging Therapies in HPV-Negative R/M HNSCC.

Treatment	Mechanism/Target	Phase	ORR (Latest)	Key Survival Outcomes
Dalpiciclib + cetuximab	CDK4/6 inhibitor plus anti-EGFR monoclonal antibody (EGFR/cell-cycle blockade)	Phase II	ORR 67.9% (95% CI 49.0–82.0)	mPFS 5.3 months; mOS 17.0 months
Palbociclib + cetuximab	CDK4/6 inhibitor plus anti-EGFR monoclonal antibody	Phase III	ORR 19% in Phase II cetuximab-resistant HPV-unrelated disease	Exploratory OS advantage in CDKN2A-altered tumors (HR 0.38; OS 9.7 vs. 4.6 mos)
Duvelisib + docetaxel	PI3Kδ/γ inhibitor plus taxane chemotherapy	Phase II	ORR 19%	mPFS 2.8 months; mOS 10.2 months
Ficlatuzumab + cetuximab	Anti-HGF monoclonal antibody plus anti-EGFR monoclonal antibody (dual pathway inhibition)	Phase III	ORR 38% in the HPV-negative subgroup in Phase II	mPFS 4.1 months; mOS 7.4 months in HPV-negative subgroup
NT219 + cetuximab	IRS1/2 degrader and STAT3 inhibitor plus anti-EGFR monoclonal antibody	Phase I/II	ORR 33% in higher-dose cohorts	DCR 67%
Afatinib + cetuximab	Pan-HER tyrosine kinase inhibitor plus anti-EGFR monoclonal antibody (vertical ErbB blockade)	Phase II	ORR 23.4%	mPFS 3.8 months; mOS 7.5 months

**Table 2:** Emerging Therapies in HPV-Positive R/M HNSCC.

Treatment	Mechanism/Target	Phase	ORR (Latest)	Key Survival Outcomes
FLX475 + pembrolizumab	CCR4 antagonist blocking Treg recruitment + anti-PD-1 checkpoint inhibitor	Phase II	ORR 22.2% in HPV-positive cohort	Generally well tolerated; reversible QT prolongation is manageable by dose reduction
ISA101b + cemiplimab	Synthetic long-peptide vaccine targeting HPV16 E6/E7 plus anti-PD-1	Phase II	ORR 6.3% overall (11.5% in stage 1); CBR 56.3%	mOS 11.3 months (53); manageable grade 3 AEs with no grade 4–5 events
CUE-101	HPV16 E7-pHLA-IL2-Fc fusion protein selectively expanding antigen-specific CD8+ T cells	Phase I/II	Clinical benefit rate 37% (1 PR and 6 durable SD ≥ 12 weeks)	mOS 24.4 months in a heavily pretreated cohort
Ozuriftamab vedotin (BA3021)	ROR2-targeted conditionally active biologic ADC delivering MMAE	Phase II	ORR 32% overall (including 1 CR)	HPV-associated subset: mPFS 4.8 months; mOS 11.6 months

**Table 3:** Emerging Therapies in HPV-Neutral R/M HNSCC.

Treatment	Mechanism/Target	Phase	ORR (Latest)	Key Survival Outcomes
Paclitaxel + cetuximab	Taxane chemotherapy plus anti-EGFR monoclonal antibody	Phase II	ORR 69.6% (Japan) and 47.4% (PACE-ACE)	mOS 13.3 months (Japan) and 14.0 months (PACE-ACE)
FID-007 (nanoparticle paclitaxel)	Nanoparticle paclitaxel with PEOX excipient for tumor-selective delivery	Phase I; Phase II combo planned	ORR 56% in the HNSCC cohort	No high-grade neuropathy reported; Phase I RP2D 125 mg/m^2^
SI-B001 (izalontamab) + paclitaxel	Bispecific EGFR × HER3 antibody plus taxane chemotherapy	Phase II	ORR 64.3%	mPFS 5.6 months
Retlirafusp alfa (SHR-1701) ± chemotherapy	Bifunctional anti–PD-L1/TGF-βRII fusion protein, sometimes combined with carboplatin/nab-paclitaxel	Phase I; prospective single-arm Phase II with chemo	ORR 20% in heavily pretreated HNSCC cohort	Hematologic toxicities common; dose 30 mg/kg Q3W established
Petosemtamab (MCLA-158)	Bispecific EGFR × LGR5 antibody with enhanced ADCC/ADCP and EGFR degradation in LGR5+ cells	Phase II; Phase III	ORR 36–40.4%	mPFS ≈ 5.0 months; mOS 11.5–12.5 months
MRG003 (becotatud vedotin)	EGFR-targeted ADC delivering MMAE via a cleavable linker with bystander effect	Phase II; Phase III	ORR 43% at 2.3 mg/kg	mPFS 4.2 months; mOS 11.3 months
Tisotumab vedotin	Tissue Factor (TF)-targeted ADC delivering MMAE via a cleavable linker	Phase II	ORR 32.5% overall; 40% in 2nd–3rd line	Median DOR 5.6 months
Enfortumab vedotin	Nectin-4-targeted ADC delivering MMAE	Phase II	ORR 23.9%	DCR 56.5%; DOR 9.4 months; mOS 6.0 months
Sacituzumab govitecan	Trop-2-targeted ADC delivering SN-38 via a hydrolyzable linker	Phase II; Phase II combo planned	ORR 16% (95% CI 7–31%)	mPFS 4.1 months; mOS 9.0 months; DOR 4.8 months
Sigvotatug vedotin (SGN-B6A)	Integrin β6-targeted ADC delivering MMAE	Phase I	ORR 20–23%	DCR 61%
Bimiralisib (PQR309)	Dual pan-PI3K/mTOR inhibitor with intermittent dosing	Phase I	ORR 17% (1 PR among 6 evaluable)	mPFS 5 months; mOS 7 months
Tipifarnib	Farnesyl transferase inhibitor targeting HRAS-dependent signaling	Phase II	ORR 55% in RUN-HN; ORR 30% investigator and 20% independent in AIM-HN	mPFS 5.6 months; mOS 15.4 months in RUN-HN
MVX-ONCO-1	Personalized, cell-based vaccine (Irradiated autologous tumor cells + GM-CSF secreting allogeneic cells).	Phase I; Phase II	ORR modest	Median OS 11.4 months (95% CI, 4.4–NR) in PII; 68.8% alive at six months

## References

[1] Fasano M., Della Corte C.M., Viscardi G., Di Liello R., Paragliola F., Sparano F., Iacovino M.L., Castrichino A., Doria F., Sica A. (2021). Head and neck cancer: The role of anti-EGFR agents in the era of immunotherapy. Ther. Adv. Med. Oncol..

[2] Kok V.C. (2020). Current Understanding of the Mechanisms Underlying Immune Evasion From PD-1/PD-L1 Immune Checkpoint Blockade in Head and Neck Cancer. Front. Oncol..

[3] Siano M., Infante G., Resteghini C., Cau M.C., Alfieri S., Bergamini C., Granata R., Miceli R., Locati L., Licitra L. (2017). Outcome of recurrent and metastatic head and neck squamous cell cancer patients after first line platinum and cetuximab therapy. Oral Oncol..

[4] Cho B.C., Braña I., Cirauqui B., Aksoy S., Couture F., Hong R.-L., Miller W.H., Chaves-Conde M., Teixeira M., Leopold L. (2024). Pembrolizumab plus epacadostat in patients with recurrent/metastatic head and neck squamous cell carcinoma (KEYNOTE-669/ECHO-304): A phase 3, randomized, open-label study. BMC Cancer.

[5] Yilmaz E., Ismaila N., Bauman J.E., Dabney R., Gan G., Jordan R., Kaufman M., Kirtane K., McBride S.M., Old M.O. (2023). Immunotherapy and Biomarker Testing in Recurrent and Metastatic Head and Neck Cancers: ASCO Guideline. J. Clin. Oncol..

[6] Tahara M., Lim D.W.-T., Keam B., Ma B., Zhang L., Wang C., Guo Y. (2025). Management approaches for recurrent or metastatic head and neck squamous cell carcinoma after anti-PD-1/PD-L1 immunotherapy. Cancer Treat. Rev..

[7] Koyama T., Kiyota N., Boku S., Imamura Y., Shibata N., Satake H., Tanaka K., Hayashi H., Onoe T., Asada Y. (2024). A phase II trial of paclitaxel plus biweekly cetuximab for patients with recurrent or metastatic head and neck cancer previously treated with both platinum-based chemotherapy and anti-PD-1 antibody. ESMO Open.

[8] Park J.C., Ahn J.S., Merkin R., Patel M., Wirth L., Roberts T.J. (2025). Correlates of Cetuximab Efficacy in Recurrent and Metastatic Head and Neck Squamous Cell Carcinoma Previously Treated With Immunotherapy. JCO Precis. Oncol..

[9] National Comprehensive Cancer Network (2025). NCCN Clinical Practice Guidelines in Oncology (NCCN Guidelines®): Head and Neck Cancers, Version 2.2025.

[10] Machiels J.-P., Leemans C.R., Golusinski W., Grau C., Licitra L., Gregoire V. (2020). Squamous cell carcinoma of the oral cavity, larynx, oropharynx and hypopharynx: EHNS–ESMO–ESTRO clinical practice guidelines for diagnosis, treatment and follow-up. Ann. Oncol..

[11] Merck & Co., Inc (2025). Merck’s KEYTRUDA® (pembrolizumab) Met Primary Endpoint of Event-Free Survival (EFS) as Perioperative Treatment Regimen in Patients with Resected, Locally Advanced Head and Neck Squamous Cell Carcinoma. https://www.merck.com/news/mercks-keytruda-pembrolizumab-met-primary-endpoint-of-event-free-survival-efs-as-perioperative-treatment-regimen-in-patients-with-resected-locally-advanced-head-and-neck-squamous-c/.

[12] BioSpace (2025). GORTEC Announces New Trial Success for Head and Neck Cancer Treatment. https://www.biospace.com/press-releases/gortec-announces-new-trial-success-for-head-and-neck-cancer-treatment.

[13] Vermorken J.B., Trigo J., Hitt R., Koralewski P., Diaz-Rubio E., Rolland F., Knecht R., Amellal N., Schueler A., Baselga J. (2007). Open-label, uncontrolled, multicenter phase II study to evaluate the efficacy and toxicity of cetuximab as a single agent in patients with recurrent and/or metastatic squamous cell carcinoma of the head and neck who failed to respond to platinum-based therapy. J. Clin. Oncol..

[14] Cohen E.E.W., Le Tourneau C., Licitra L., Ahn M.-J., Soria A., Machiels J.-P., Mach N., Mehra R., Burtness B., Zhang P. (2019). Pembrolizumab versus methotrexate, docetaxel, or cetuximab for recurrent or metastatic head-and-neck squamous cell carcinoma (KEYNOTE-040): A randomised, open-label, phase 3 study. Lancet.

[15] Ferris R.L., Blumenschein G., Fayette J., Guigay J., Colevas A.D., Licitra L., Harrington K., Kasper S., Vokes E.E., Even C. (2016). Nivolumab for recurrent squamous-cell carcinoma of the head and neck. N. Engl. J. Med..

[16] Fayette J., Harrington K., Siu L.L., Liu Y.-C., Tahara M., Machiels J.-P., Rischin D., Seiwert T.Y., Ferris R.L., Keilholz U. (2025). INTERLINK-1: A Phase III, Randomized, Placebo-Controlled Study of Monalizumab plus Cetuximab in Recurrent/Metastatic Head and Neck Squamous Cell Carcinoma. Clin. Cancer Res..

[17] Burcher K.M., Bloomer C.H., Gavrila E., Kalada J.M., Chang M.J., Gebeyehu R.R., Song A.H., Khoury L.M., Lycan T.W., Kinney R. (2024). Study protocol: Phase II study to evaluate the effect of cetuximab monotherapy after immunotherapy with PD-1 inhibitors in patients with head and neck squamous cell cancer. Ther. Adv. Med. Oncol..

[18] Fuereder T., Klinghammer K., Hahn D., Grünberger B., Melchardt T., Greil R., Kocher F., Gamerith G., Wagner C., Berchtold L. (2024). 880P Paclitaxel plus cetuximab for the treatment of recurrent and/or metastatic head and neck cancer after first-line checkpoint inhibitor failure: Primary analysis from the pace ace trial. Ann. Oncol..

[19] Chow L.D., Hsu R., Nieva J.J., Umayam R., Bryant A.S., Tsao-Wei D., Hsieh M., Yin R., El-Khoueiry A.B., Thomas J.S. (2024). Efficacy from the phase 1 study of FID-007, a novel nanoparticle paclitaxel formulation, in patients with head and neck squamous cell carcinoma. J. Clin. Oncol..

[20] Fulgent Pharma LLC (2024). A Phase 2, Randomized, Multicenter, Open-label, Study of FID-007 in Combination with Cetuximab in Patients with Advanced Head and Neck Squamous Cell Carcinoma.

[21] Xue J., Kong D., Yao Y., Yang L., Yao Q., Zhu Y., Ding Y., Yang F., Gong J., Shen L. (2020). Prediction of Human Pharmacokinetics and Clinical Effective Dose of SI-B001, an EGFR/HER3 Bi-specific Monoclonal Antibody. J. Pharm. Sci..

[22] Xue J., Ma Y., Zhao Y., Wang Y., Hong W., Huang Y., Yang Y., Fang W., Hong S., Zhang Y. (2025). Izalontamab (SI-B001), a novel EGFRxHER3 bispecific antibody in patients with Locally Advanced or Metastatic Epithelial Tumor: Results from first-in-human phase I/Ib study. Clin. Cancer Res..

[23] Xue L., Yang K., Fang M., Ma X., Zou W., Ding M., Wang Z., Peng Y., Xiao S., Wang H. (2023). Results from two phase II studies of SI-B001, an EGFR×HER3 bispecific antibody, with/without chemotherapy in patients with recurrent and metastatic head and neck squamous cell carcinoma (HNSCC). J. Clin. Oncol..

[24] Mariathasan S., Turley S.J., Nickles D., Castiglioni A., Yuen K., Wang Y., Kadel E.E., Koeppen H., Astarita J.L., Cubas R. (2018). TGFbeta attenuates tumour response to PD-L1 blockade by contributing to exclusion of T cells. Nature.

[25] Liu D., Zhou J., Wang Y., Li M., Jiang H., Liu Y., Yin X., Ge M., Xiang X., Ying J. (2022). Bifunctional anti-PD-L1/TGF-betaRII agent SHR-1701 in advanced solid tumors: A dose-escalation, dose-expansion, and clinical-expansion phase 1 trial. BMC Med..

[26] Ji D., Liu X., Guo Y., Yang Y., Sang Y., Chen G., Dong S., Wang Y., He X., Ying H. (2025). Retlirafusp alfa-a bifunctional anti-PD-L1/TGF-bRII agent plus nab-paclitaxel and carboplatin in pre-treated recurrent/metastatic head and neck squamous cell carcinoma (R/M HNSCC): A prospective, single-arm, phase II clinical trial. J. Clin. Oncol..

[27] Brockstedt D.G., Grant A., Adamik J., Trujillo D., Goyal R.K., Ho W., Ikeda S., Zhu Q., Anders R.A., Sabouri M. (2023). Clinical and biological activity of FLX475, an oral CCR4 antagonist, in advanced cancer. J. Clin. Oncol..

[28] Muzaffar J., Kirtane K., Redman R., Yang M.-H., Kim T.M., Liu S., Lynch R., Brahmer J., LoRusso P., Henick B. (2024). Abstract CT226: Phase 2 study of oral CCR4 antagonist FLX475 (tivumecirnon) plus pembrolizumab in subjects with head and neck squamous cell carcinoma (HNSCC) previously treated with checkpoint inhibitor. Cancer Res..

[29] Riess C., Irmscher N., Salewski I., Strüder D., Classen C.-F., Große-Thie C., Junghanss C., Maletzki C. (2020). Cyclin-dependent kinase inhibitors in head and neck cancer and glioblastoma-backbone or add-on in immune-oncology?. Cancer Metastasis Rev..

[30] Seront E.E.A. (2019). Phase 1 study evaluating the association of the cyclin D1-CDK4/6 complex and the EGFR/AKT pathway in squamous cell carcinoma of the head and neck. Front. Oncol..

[31] Network C.G.A. (2015). Comprehensive genomic characterization of head and neck squamous cell carcinomas. Nature.

[32] Zahavi D.J., Weiner L.M. (2019). Targeting Multiple Receptors to Increase Checkpoint Blockade Efficacy. Int. J. Mol. Sci..

[33] Smeets S.J., Braakhuis B.J.M., Abbas S., Snijders P.J.F., Ylstra B., A van de Wiel M., Meijer G.A., Leemans C.R., Brakenhoff R.H. (2005). Genome-wide DNA copy number alterations in head and neck squamous cell carcinomas with or without oncogene-expressing human papillomavirus. Oncogene.

[34] Scantlebury J.B., Luo J., Thorstad W.L., El-Mofty S.K., Lewis J.S. (2013). Cyclin D1-a prognostic marker in oropharyngeal squamous cell carcinoma that is tightly associated with high-risk human papillomavirus status. Hum. Pathol..

[35] Reed A.L., Califano J., Cairns P., Westra W.H., Jones R.M., Koch W., Ahrendt S., Eby Y., Sewell D., Nawroz H. (1996). High frequency of p16 (CDKN2/MTS-1/INK4A) inactivation in head and neck squamous cell carcinoma. Cancer Res..

[36] Beck T.N., Georgopoulos R., Shagisultanova E.I., Sarcu D., Handorf E.A., Dubyk C., Lango M.N., Ridge J.A., Astsaturov I., Serebriiskii I.G. (2016). EGFR and RB1 as Dual Biomarkers in HPV-Negative Head and Neck Cancer. Mol. Cancer Ther..

[37] Kalish L.H., Kwong R.A., Cole I.E., Gallagher R.M., Sutherland R.L., Musgrove E.A. (2004). Deregulated cyclin D1 expression is associated with decreased efficacy of the selective epidermal growth factor receptor tyrosine kinase inhibitor gefitinib in head and neck squamous cell carcinoma cell lines. Clin. Cancer Res..

[38] Houyu J., Wu Y., Shi C., Chen L., Sun L., Xia R., Sun S., Hu J., He Y., Ren G. (2024). 881P Cetuximab with dalpicilib in patients with HPV-negative, anti-PD-1 resistant R/M HNSCC. Ann. Oncol..

[39] Ju H., Hu J., Wu Y., Xia R.-H., Shi C., Ren G., Hu Y. (2025). Cetuximab plus dalpiciclib in patients with HPV-negative, anti-PD-1-resistant recurrent or metastatic head and neck squamous cell carcinoma. J. Clin. Oncol..

[40] Adkins D., Ley J.B., Cohen J., Oppelt P. (2022). The Potential for Selective Cyclin-Dependent Kinase 4/6 Inhibition in the Therapy for Head and Neck Squamous Cell Carcinoma. Cancer J..

[41] Worden F.P., Pisick E., Rothe M., Mangat P.K., Garrett-Mayer E., Khalil M.F., Carrizosa D.R., Bauman J.R., Leidner R.S., Duvivier H.L. (2024). Palbociclib in Patients With Head and Neck Cancer and Other Tumors With CDKN2A Alterations: Results From the Targeted Agent and Profiling Utilization Registry Study. JCO Precis. Oncol..

[42] Michel L., Ley J., Wildes T.M., Schaffer A., Robinson A., Chun S.-E., Lee W., Lewis J., Trinkaus K., Adkins D. (2016). Phase I trial of palbociclib, a selective cyclin dependent kinase 4/6 inhibitor, in combination with cetuximab in patients with recurrent/metastatic head and neck squamous cell carcinoma. Oral Oncol..

[43] Adkins D., Ley J., Neupane P., Wordem F., Sacco A.G., Palka K., Grilley-Olson J.E., Maggiore R., Salama N.N., Trinkaus K. (2019). Palbociclib and cetuximab in platinum-resistant and in cetuximab-resistant human papillomavirus-unrelated head and neck cancer: A multicentre, multigroup, phase 2 trial. Lancet Oncol..

[44] Oppelt P., Ley J.C., Worden F., Palka K., Maggiore R., Liu J., Adkins D. (2021). Palbociclib and cetuximab in cetuximab-resistant human papillomavirus-related oropharynx squamous-cell carcinoma: A multicenter phase 2 trial. Oral Oncol..

[45] Adkins D.R., Lin J.-C., Sacco A., Ley J., Oppelt P., Vanchenko V., Komashko N., Yen C.-J., Wise-Draper T., Gonzalez J.L.-P. (2021). Palbociclib and cetuximab compared with placebo and cetuximab in platinum-resistant, cetuximab-naïve, human papillomavirus-unrelated recurrent or metastatic head and neck squamous cell carcinoma: A double-blind, randomized, phase 2 trial. Oral Oncol..

[46] Ley J.C., Cohen J., Liu J., Thomeczek B., Oppelt P.J., Adkins D. (2023). Palbociclib + cetuximab versus cetuximab in patients with CDKN2A-altered, anti-PD-1 resistant, HPV-negative head and neck squamous cell carcinoma (HNSCC): A phase 3 trial. J. Clin. Oncol..

[47] Marquard F.E., Jucker M. (2020). PI3K/AKT/mTOR signaling as a molecular target in head and neck cancer. Biochem. Pharmacol..

[48] Jung K., Kang H., Mehra R. (2018). Targeting phosphoinositide 3-kinase (PI3K) in head and neck squamous cell carcinoma (HNSCC). Cancers Head Neck.

[49] Glaviano A., Foo A.S.C., Lam H.Y., Yap K.C.H., Jacot W., Jones R.H., Eng H., Nair M.G., Makvandi P., Geoerger B. (2023). PI3K/AKT/mTOR signaling transduction pathway and targeted therapies in cancer. Mol. Cancer.

[50] Engelman J.A. (2009). Targeting PI3K signalling in cancer: Opportunities, challenges and limitations. Nat. Rev. Cancer.

[51] Peng Y., Wang Y., Zhou C., Mei W., Zeng C. (2022). PI3K/Akt/mTOR Pathway and Its Role in Cancer Therapeutics: Are We Making Headway?. Front. Oncol..

[52] Castel P., Toska E., Engelman J.A., Scaltriti M. (2021). The present and future of PI3K inhibitors for cancer therapy. Nat. Cancer.

[53] Vanhaesebroeck B., Perry M.W.D., Brown J.R., André F., Okkenhaug K. (2021). PI3K inhibitors are finally coming of age. Nat. Rev. Drug Discov..

[54] Yang J., Nie J., Ma X., Wei Y., Peng Y., Wei X. (2019). Targeting PI3K in cancer: Mechanisms and advances in clinical trials. Mol. Cancer.

[55] Alsahafi E., Begg K., Amelio I., Raulf N., Lucarelli P., Sauter T., Tavassoli M. (2019). Clinical update on head and neck cancer: Molecular biology and ongoing challenges. Cell Death Dis..

[56] Li Q., Tie Y., Alu A., Ma X., Shi H. (2023). Targeted therapy for head and neck cancer: Signaling pathways and clinical studies. Signal Transduct. Target. Ther..

[57] Beck T.N., Golemis E.A. (2016). Genomic insights into head and neck cancer. Cancers Head Neck.

[58] Grandis J.R., Tweardy D.J. (1993). Elevated levels of transforming growth factor alpha and epidermal growth factor receptor messenger RNA are early markers of carcinogenesis in head and neck cancer. Cancer Res..

[59] Smith A.E., Chan S., Wang Z., McCloskey A., Reilly Q., Wang J.Z., Patel H.V., Koshizuka K., Soifer H.S., Kessler L. (2023). Tipifarnib Potentiates the Antitumor Effects of PI3Kalpha Inhibition in PIK3CA- and HRAS-Dysregulated HNSCC via Convergent Inhibition of mTOR Activity. Cancer Res..

[60] Song M.S., Salmena L., Pandolfi P.P. (2012). The functions and regulation of the PTEN tumour suppressor. Nat. Rev. Mol. Cell Biol..

[61] Hoxhaj G.M., Brendan D. (2020). The PI3K-AKT network at the interface of oncogenic signaling and cancer metabolism. Nat. Rev. Cancer.

[62] Eze N., Lee J.-W., Yang D.-H., Zhu F., Neumeister V., Sandoval-Schaefer T., Mehra R., Ridge J.A., Forastiere A., Chung C.H. (2019). PTEN loss is associated with resistance to cetuximab in patients with head and neck squamous cell carcinoma. Oral Oncol..

[63] Zandberg D.P., Menk A.V., Velez M., Normolle D., DePeaux K., Liu A., Ferris R.L., Delgoffe G.M. (2021). Tumor hypoxia is associated with resistance to PD-1 blockade in squamous cell carcinoma of the head and neck. J. Immunother. Cancer.

[64] Hanna G.J., Oakley L.B., Shi R., Oneill A., Shin K.Y., Scarfo N., Sehgal K., Dennis M.J., Quinn N., Jo V.Y. (2024). Duvelisib with Docetaxel for Patients with Anti–PD-1 Refractory, Recurrent, or Metastatic Head and Neck Squamous Cell Carcinoma. Clin. Cancer Res..

[65] Tarantelli C., Gaudio E., Arribas A.J., Kwee I., Hillmann P., Rinaldi A., Cascione L., Spriano F., Bernasconi E., Guidetti F. (2018). PQR309 Is a Novel Dual PI3K/mTOR Inhibitor with Preclinical Antitumor Activity in Lymphomas as a Single Agent and in Combination Therapy. Clin. Cancer Res..

[66] Beaufils F., Cmiljanovic N., Cmiljanovic V., Bohnacker T., Melone A., Marone R., Jackson E., Zhang X., Sele A., Borsari C. (2017). 5-(4,6-Dimorpholino-1,3,5-triazin-2-yl)-4-(trifluoromethyl)pyridin-2-amine (PQR309), a Potent, Brain-Penetrant, Orally Bioavailable, Pan-Class I PI3K/mTOR Inhibitor as Clinical Candidate in Oncology. J. Med. Chem..

[67] Johnson F.M., Janku F., Gouda M.A., Tran H.T., Kawedia J.D., Schmitz D., Streefkerk H., Lee J.J., Andersen C.R., Deng D. (2022). Inhibition of the Phosphatidylinositol-3 Kinase Pathway Using Bimiralisib in Loss-of-Function NOTCH1-Mutant Head and Neck Cancer. Oncologist.

[68] Janku F., Choong G.M., Opyrchal M., Dowlati A., Hierro C., Rodon J., Wicki A., Forster M.D., Blagden S.P., Yin J. (2024). A Phase I Study of the Oral Dual-Acting Pan-PI3K/mTOR Inhibitor Bimiralisib in Patients with Advanced Solid Tumors. Cancers.

[69] Schinke H., Shi E., Lin Z., Quadt T., Kranz G., Zhou J., Wang H., Hess J., Heuer S., Belka C. (2022). A transcriptomic map of EGFR-induced epithelial-to-mesenchymal transition identifies prognostic and therapeutic targets for head and neck cancer. Mol. Cancer.

[70] Mountzios G., Rampias T., Psyrri A. (2014). The mutational spectrum of squamous-cell carcinoma of the head and neck: Targetable genetic events and clinical impact. Ann. Oncol..

[71] Yahia H.B., Petit F.M., Saada-Bouzid E. (2023). Targeting Harvey rat sarcoma viral oncogene homolog in head and neck cancer: How to move forward?. Curr. Opin. Oncol..

[72] Whyte D.B., Kirschmeier P., Hockenberry T.N., Nunez-Oliva I., James L., Catino J.J., Bishop W.R., Pai J.-K. (1997). K- and N-Ras are geranylgeranylated in cells treated with farnesyl protein transferase inhibitors. J. Biol. Chem..

[73] Cox A.D., Der C.J., Philips M.R. (2015). Targeting RAS Membrane Association: Back to the Future for Anti-RAS Drug Discovery?. Clin. Cancer Res..

[74] Gilardi M., Wang Z., Proietto M., Chillà A., Calleja-Valera J.L., Goto Y., Vanoni M., Janes M.R., Mikulski Z., Gualberto A. (2020). Tipifarnib as a Precision Therapy for HRAS-Mutant Head and Neck Squamous Cell Carcinomas. Mol. Cancer Ther..

[75] Ho A.L., Brana I., Haddad R., Bauman J., Bible K., Oosting S., Wong D.J., Ahn M.-J., Boni V., Even C. (2021). Tipifarnib in Head and Neck Squamous Cell Carcinoma With *HRAS* Mutations. J. Clin. Oncol..

[76] Haddad R.I., Adkins D., Licitra L.F., Bruce J.Y., Gillison M.L., Ahn M.-J., Hsieh C.-Y., Wang H.-M., Psyrri A., Machiels J.-P.H. (2021). The AIM-HN Study: A pivotal study evaluating the efficacy of tipifarnib in patients with recurrent or metastatic head and neck squamous cell carcinoma with HRAS mutations. J. Clin. Oncol..

[77] Ho A., Adkins D., Hanna G., Bruce J., Ahn M.-J., Docampo L.I., Kang H., Wong D., Psyrri A., Gillison M. (2023). LBA47 A phase II study evaluating tipifarnib in mHRAS, recurrent or metastatic head and neck squamous cell carcinoma (HNSCC) (AIM-HN study). Ann. Oncol..

[78] Jagadeeshan S., Suryamohan K., Shin N., Mathukkada S., Boyko A., Melikhova D., Tsareva A., Yunusova L., Pravdivtseva E., Stupichev D. (2024). Evolutionary dynamics of tipifarnib in HRAS mutated head and neck squamous cell carcinoma. Oral Oncol..

[79] Kura Oncology, Inc (2021). Combination Trial of Tipifarnib and Alpelisib in Adult Recurrent/Metastatic Head and Neck Squamous Cell Carcinoma (R/M HNSCC).

[80] Shah N., Trivedi T., Tankshali R., Goswami J., Jetly D., Kobawala T., Shukla S., Shah P., Verma R. (2006). Stat3 Expression in Oral Squamous Cell Carcinoma: Association with Clinicopathological Parameters and Survival. Int. J. Biol. Markers.

[81] Rosenberg A.J., Kato S., Johnson D., Popovtzer A., Chiu V.K., Geva R., Ben-David H., Meirson T., Schickler M., Reuveni H. (2024). Abstract 5181: Early activity and biomarker evaluation of NT219 in combination with cetuximab in a Phase 1/2 study of recurrent/metastatic squamous cell carcinoma of the head and neck (R/M SCCHN). Cancer Res..

[82] Lee T.L., Yeh J., Van Waes C., Chen Z. (2006). Epigenetic modification of SOCS-1 differentially regulates STAT3 activation in response to interleukin-6 receptor and epidermal growth factor receptor signaling through JAK and/or MEK in head and neck squamous cell carcinomas. Mol. Cancer Ther..

[83] Masuda M., Suzui M., Yasumatu R., Nakashima T., Kuratomi Y., Azuma K., Tomita K., Komiyama S., Weinstein I.B. (2002). Constitutive activation of signal transducers and activators of transcription 3 correlates with cyclin D1 overexpression and may provide a novel prognostic marker in head and neck squamous cell carcinoma. Cancer Res..

[84] Gaykalova D.A., Manola J.B., Ozawa H., Zizkova V., Morton K., Bishop J.A., Sharma R., Zhang C., Michailidi C., Considine M. (2015). NF-κB and stat3 transcription factor signatures differentiate HPV-positive and HPV-negative head and neck squamous cell carcinoma. Int. J. Cancer.

[85] Dale O.T., Aleksic T., Shah K.A., Han C., Mehanna H., Rapozo D.C., Sheard J.D.H., Goodyear P., Upile N.S., Robinson M. (2015). IGF-1R expression is associated with HPV-negative status and adverse survival in head and neck squamous cell carcinoma. Carcinogenesis.

[86] Rosenberg A., Kato S., Johnson D., Popovtzer A., Chiu V., Geva R., Schickler M., Reuveni H. (2024). 27O Interim results of a phase I/II trial of NT219 in combination with cetuximab in patients with advanced/metastatic squamous cell carcinoma of the head and neck (SCCHN). ESMO Open.

[87] Kalyankrishna S., Grandis J.R. (2006). Epidermal growth factor receptor biology in head and neck cancer. J. Clin. Oncol..

[88] Chakravarti A., Loeffler J.S., Dyson N.J. (2002). Insulin-like growth factor receptor I mediates resistance to anti-epidermal growth factor receptor therapy in primary human glioblastoma cells through continued activation of phosphoinositide 3-kinase signaling. Cancer Res..

[89] Erjala K., Sundvall M., Junttila T.T., Zhang N., Savisalo M., Mali P., Kulmala J., Pulkkinen J., Grenman R., Elenius K. (2006). Signaling via erbB2 and erbB3 associates with resistance and epidermal growth factor receptor (EGFR) amplification with sensitivity to EGFR inhibitor gefitinib in head and neck squamous cell carcinoma cells. Clin. Cancer Res..

[90] Saddawi-Konefka R., Schokrpur S., Lui A.J., Gutkind J.S. (2022). HER2 and HER3 as Therapeutic Targets in Head and Neck Cancer. Cancer J..

[91] Gazzah A., Boni V., Soria J.-C., Calles A., Even C., Doger B., Mahjoubi L., Bahleda R., Ould-Kaci M., Esler A. (2018). A phase 1b study of afatinib in combination with standard-dose cetuximab in patients with advanced solid tumours. Eur. J. Cancer.

[92] Yonesaka K., Tanaka K., Kitano M., Kawakami H., Hayashi H., Takeda M., Sakai K., Nishio K., Doi K., Nakagawa K. (2019). Aberrant HER3 ligand heregulin-expressing head and neck squamous cell carcinoma is resistant to anti-EGFR antibody cetuximab, but not second-generation EGFR-TKI. Oncogenesis.

[93] Bhatia A.K., Wei W., Chiorazzi M., Deshpande H.A., Reynolds J., Gehan D., Tara H.H., Newton B.R., Verma A., Sayed Z. (2025). Phase 2 trial of dual EGFR inhibition with cetuximab and afatinib in patients with recurrent/metastatic head and neck squamous cell cancers (HNSCC). J. Clin. Oncol..

[94] Bhatia A., Mehra R., Bauman J., Khan S.A., Wei W., Neumeister V., Sandoval-Schaefer T., Alpaugh R.K., Lango M., Rimm D.L. (2025). Phase II Trial of Chemotherapy, Cetuximab, and Erlotinib in Patients With Metastatic or Recurrent Squamous Cell Carcinoma of the Head and Neck. Head Neck.

[95] Machiels J.-P.H., I Haddad R., Fayette J., Licitra L.F., Tahara M., Vermorken J.B., Clement P.M., Gauler T., Cupissol D., Grau J.J. (2015). Afatinib versus methotrexate as second-line treatment in patients with recurrent or metastatic squamous-cell carcinoma of the head and neck progressing on or after platinum-based therapy (LUX-Head & Neck 1): An open-label, randomised phase 3 trial. Lancet Oncol..

[96] van de Donk N.W.C.J., Zweegman S. (2023). T-cell-engaging bispecific antibodies in cancer. Lancet.

[97] Paul S., Konig M.F., Pardoll D.M., Bettegowda C., Papadopoulos N., Wright K.M., Gabelli S.B., Ho M., van Elsas A., Zhou S. (2024). Cancer therapy with antibodies. Nat. Rev. Cancer.

[98] Dalley A.J., Majeed A.A.A., Pitty L.P., Major A.G., Farah C.S. (2015). LGR5 expression in oral epithelial dysplasia and oral squamous cell carcinoma. Oral Surg. Oral Med. Oral Pathol. Oral Radiol..

[99] Herpers B., Eppink B., James M.I., Cortina C., Cañellas-Socias A., Boj S.F., Hernando-Momblona X., Glodzik D., Roovers R.C., van de Wetering M. (2022). Functional patient-derived organoid screenings identify MCLA-158 as a therapeutic EGFR x LGR5 bispecific antibody with efficacy in epithelial tumors. Nat. Cancer.

[100] Lundberg A.S., Geuijen C.A.W., Hill S., van Bueren J.J.L., Fumagalli A., de Kruif J., Silverman P.B., Tabernero J. (2025). Petosemtamab, a Bispecific Antibody Targeting Epidermal Growth Factor Receptor (EGFR) and Leucine-Rich G Repeat-Containing Protein-Coupled Receptor (LGR5) Designed for Broad Clinical Applications. Cancers.

[101] Le Tourneau C., Fayette J., Even C., Sacco A., Daste A., Braña I., van Herpen C., Mazard T., Henry S., Saerens M. (2024). 411MO Petosemtamab (MCLA-158) monotherapy in previously treated (2L+) recurrent/metastatic (r/m) head and neck squamous cell carcinoma (HNSCC): Phase II trial. Ann. Oncol..

[102] Machiels J.-P., Fayette J., Haddad R., Adkins D., Gillison M., Harrington K.J., Kim S.-B., Le Tourneau C., Psyrri A., Rosenberg A. (2025). LiGeR-HN phase III trials of petosemtamab + pembrolizumab and petosemtamab monotherapy in recurrent or metastatic HNSCC. Futur. Oncol..

[103] Haddad R., Gillison M., Harrington K., Kim S.-B., Le Tourneau C., Rosenberg A., Ford J., Shen Y.-M., Yao D., Zohren F. (2024). 943TiP Petosemtamab compared with investigator’s choice monotherapy in previously treated patients (pts) with recurrent/metastatic (r/m) head and neck squamous cell carcinoma (HNSCC): A randomized, open-label, phase III trial. Ann. Oncol..

[104] Xu H., Stabile L.P., Gubish C.T., Gooding W.E., Grandis J.R., Siegfried J.M. (2011). Dual blockade of EGFR and c-Met abrogates redundant signaling and proliferation in head and neck carcinoma cells. Clin. Cancer Res..

[105] Rothenberger N.J., Stabile L.P. (2017). Hepatocyte Growth Factor/c-Met Signaling in Head and Neck Cancer and Implications for Treatment. Cancers.

[106] Kumar D., Kandl C., Hamilton C.D., Shnayder Y., Tsue T.T., Kakarala K., Ledgerwood L., Sun X.S., Huang H.J., Girod D. (2015). Mitigation of Tumor-Associated Fibroblast-Facilitated Head and Neck Cancer Progression With Anti-Hepatocyte Growth Factor Antibody Ficlatuzumab. JAMA Otolaryngol. Head Neck Surg..

[107] Centuori S.M., Bauman J.E. (2022). c-Met Signaling as a Therapeutic Target in Head and Neck Cancer. Cancer J..

[108] Mandal M., Myers J.N., Lippman S.M., Johnson F.M., Williams M.D., Rayala S., Ohshiro K., Rosenthal D.I., Weber R.S., Gallick G.E. (2008). Epithelial to mesenchymal transition in head and neck squamous carcinoma. Cancer.

[109] Bauman J.E., Saba N.F., Roe D., Bauman J.R., Kaczmar J., Bhatia A., Muzaffar J., Julian R., Wang S., Bearelly S. (2023). Randomized Phase II Trial of Ficlatuzumab With or Without Cetuximab in Pan-Refractory, Recurrent/Metastatic Head and Neck Cancer. J. Clin. Oncol..

[110] Bauman J.E., Ohr J., Gooding W.E., Ferris R.L., Duvvuri U., Kim S., Johnson J.T., Soloff A.C., Wallweber G., Winslow J. (2020). Phase I Study of Ficlatuzumab and Cetuximab in Cetuximab-Resistant, Recurrent/Metastatic Head and Neck Cancer. Cancers.

[111] Bauman J., Allman K., Haddad R. (2024). FIERCE-HN: A Multicenter, Randomized, Placebo-controlled, Phase 3 Study of ficlatuzumab + cetuximab in pts w/ recurrent or Metastatic (R/M) HPV-negative Head and Neck Squamous Cell Carcinoma (HNSCC). Int. J. Radiat. Oncol..

[112] Gogia P., Ashraf H., Bhasin S., Xu Y. (2023). Antibody-Drug Conjugates: A Review of Approved Drugs and Their Clinical Level of Evidence. Cancers.

[113] Shastry M., Gupta A., Chandarlapaty S., Young M., Powles T., Hamilton E. (2023). Rise of Antibody-Drug Conjugates: The Present and Future. Am. Soc. Clin. Oncol. Educ. Book.

[114] Qiu M.-Z., Zhang Y., Guo Y., Guo W., Nian W., Liao W., Xu Z., Zhang W., Zhao H.-Y., Wei X. (2022). Evaluation of Safety of Treatment With Anti–Epidermal Growth Factor Receptor Antibody Drug Conjugate MRG003 in Patients With Advanced Solid Tumors. JAMA Oncol..

[115] Xue L., Han Y., Zhang Q., Li X., Fang M., Zhong L., Wang S., Liu Y., Zhang S., Guo Y. (2023). 939P Efficacy and safety of a novel anti-EGFR ADC MRG003 in recurrent or metastatic squamous cell carcinoma of the head and neck patients. Ann. Oncol..

[116] Shanghai Miracogen Inc (2023). A Study to Evaluate MRG003 vs. Cetuximab/Methotrexate in the Treatment of Patients with RM-SCCHN.

[117] de Goeij B.E., Satijn D., Freitag C.M., Wubbolts R., Bleeker W.K., Khasanov A., Zhu T., Chen G., Miao D., van Berkel P.H. (2015). High Turnover of Tissue Factor Enables Efficient Intracellular Delivery of Antibody–Drug Conjugates. Mol. Cancer Ther..

[118] Theunissen J.-W., Cai A.G., Bhatti M.M., Cooper A.B., Avery A.D., Dorfman R., Guelman S., Levashova Z., Migone T.-S. (2018). Treating Tissue Factor-Positive Cancers with Antibody-Drug Conjugates That Do Not Affect Blood Clotting. Mol. Cancer Ther..

[119] Smith S.A., Travers R.J., Morrissey J.H. (2015). How it all starts: Initiation of the clotting cascade. Crit. Rev. Biochem. Mol. Biol..

[120] Grover S.P., Mackman N. (2018). Tissue Factor: An Essential Mediator of Hemostasis and Trigger of Thrombosis. Arterioscler. Thromb. Vasc. Biol..

[121] van den Berg Y.W., Osanto S., Reitsma P.H., Versteeg H. (2012). The relationship between tissue factor and cancer progression: Insights from bench and bedside. Blood.

[122] Unruh D., Horbinski C. (2020). Beyond thrombosis: The impact of tissue factor signaling in cancer. J. Hematol. Oncol..

[123] Doronina S., Toki B.E., Torgov M.Y., Mendelsohn B.A., Cerveny C.G., Chace D.F., DeBlanc R.L., Gearing R.P., Bovee T.D., Siegall C.B. (2003). Development of potent monoclonal antibody auristatin conjugates for cancer therapy. Nat. Biotechnol..

[124] Koga Y., Manabe S., Aihara Y., Sato R., Tsumura R., Iwafuji H., Furuya F., Fuchigami H., Fujiwara Y., Hisada Y. (2015). Antitumor effect of antitissue factor antibody-MMAE conjugate in human pancreatic tumor xenografts. Int. J. Cancer.

[125] Bakema J.E., Walsum M.S.-V., Harris J.R., Ganzevles S.H., Muthuswamy A., Houtkamp M., Plantinga T.S., Bloemena E., Brakenhoff R.H., Breij E.C. (2024). An Antibody-Drug Conjugate Directed to Tissue Factor Shows Preclinical Antitumor Activity in Head and Neck Cancer as a Single Agent and in Combination with Chemoradiotherapy. Mol. Cancer Ther..

[126] Bogani G., Coleman R.L., Vergote I., Raspagliesi F., Lorusso D., Monk B.J. (2023). Tisotumab vedotin in recurrent or metastatic cervical cancer. Curr. Probl. Cancer.

[127] Shanghai Miracogen Inc (2018). Efficacy and Safety Study of Tisotumab Vedotin for Patients with Solid Tumors (innovaTV 207).

[128] Sun L., Fayette J., Salas S., Hong D.S., Adkins D., Dunn L., Ciardiello F., Cirauqui B., William W.N., Saba N.F. (2024). Tisotumab Vedotin in Head and Neck Squamous Cell Carcinoma: Updated Analysis from innovaTV 207 Part C. J. Clin. Oncol..

[129] Sanders C., Lau J.-F., Dietrich D., Strieth S., Brossart P., Kristiansen G. (2022). Nectin-4 is widely expressed in head and neck squamous cell carcinoma. Oncotarget.

[130] Challita-Eid P.M., Satpayev D., Yang P., An Z., Morrison K., Shostak Y., Raitano A., Nadell R., Liu W., Lortie D.R. (2016). Enfortumab Vedotin Antibody-Drug Conjugate Targeting Nectin-4 Is a Highly Potent Therapeutic Agent in Multiple Preclinical Cancer Models. Cancer Res..

[131] Olson D., Younan P., Liu B., Blahnik-Fagan G., Gosink J., Snead K., Tenn E., Hensley K., Sahetya D., Nesterova A. (2022). 1187 Enfortumab vedotin induces immunogenic cell death, elicits antitumor immune memory, and shows enhanced preclinical activity in combination with immune checkpoint inhibitors. J. Immunother. Cancer.

[132] Rosenberg J., Sridhar S.S., Zhang J., Smith D., Ruether D., Flaig T.W., Baranda J., Lang J., Plimack E.R., Sangha R. (2020). EV-101: A Phase I Study of Single-Agent Enfortumab Vedotin in Patients With Nectin-4-Positive Solid Tumors, Including Metastatic Urothelial Carcinoma. J. Clin. Oncol..

[133] Swiecicki P.L., Yilmaz E., Rosenberg A.J., Fujisawa T., Bruce J.Y., Meng C., Wozniak M., Zhao Y., Mihm M., Kaplan J. (2025). Phase II Trial of Enfortumab Vedotin in Patients With Previously Treated Advanced Head and Neck Cancer. J. Clin. Oncol..

[134] Swiecicki P., Yilmaz E., Rosenberg A., Fujisawa T., Bruce J., Meng C., Wozniak M., Wang L., Gorla S., Geiger J. (2024). Enfortumab Vedotin (EV) in the Previously Treated Advanced Head and Neck Cancer (HNC) Cohort of EV-202. Int. J. Radiat. Oncol..

[135] Goldenberg D.M., Cardillo T.M., Govindan S.V., Rossi E.A., Sharkey R.M. (2015). Trop-2 is a novel target for solid cancer therapy with sacituzumab govitecan (IMMU-132), an antibody-drug conjugate (ADC). Oncotarget.

[136] Goldenberg D.M., Sharkey R.M. (2019). Antibody-drug conjugates targeting TROP-2 and incorporating SN-38: A case study of anti-TROP-2 sacituzumab govitecan. MAbs.

[137] Cardillo T.M., Govindan S.V., Sharkey R.M., Trisal P., Goldenberg D.M. (2011). Humanized anti-Trop-2 IgG-SN-38 conjugate for effective treatment of diverse epithelial cancers: Preclinical studies in human cancer xenograft models and monkeys. Clin. Cancer Res..

[138] Starodub A.N., Ocean A.J., Shah M.A., Guarino M.J., Picozzi V.J., Vahdat L.T., Thomas S.S., Govindan S.V., Maliakal P.P., Wegener W.A. (2015). First-in-Human Trial of a Novel Anti-Trop-2 Antibody-SN-38 Conjugate, Sacituzumab Govitecan, for the Treatment of Diverse Metastatic Solid Tumors. Clin. Cancer Res..

[139] Liu X., Ma L., Li J., Sun L., Yang Y., Liu T., Xing D., Yan S., Zhang M. (2024). Trop2-targeted therapies in solid tumors: Advances and future directions. Theranostics.

[140] Bardia A., Messersmith W., Kio E., Berlin J., Vahdat L., Masters G., Moroose R., Santin A., Kalinsky K., Picozzi V. (2021). Sacituzumab govitecan, a Trop-2-directed antibody-drug conjugate, for patients with epithelial cancer: Final safety and efficacy results from the phase I/II IMMU-132-01 basket trial. Ann. Oncol..

[141] Michel L., Jimeno A., Sukari A., Beck J.T., Chiu J., Ahern E., Hilton J., Even C., Zanetta S., Mekan S. (2024). Sacituzumab Govitecan in Patients with Relapsed/Refractory Advanced Head and Neck Squamous Cell Carcinoma: Results from the Phase II TROPiCS-03 Basket Study. Clin. Cancer Res..

[142] Gilead Sciences, Inc (2025). A Study of Sacituzumab Govitecan in Combination with Cetuximab in People with Head and Neck Squamous Cell Cancer (HNSCC).

[143] Lyon R.P., Jonas M., Frantz C., Trueblood E.S., Yumul R., Westendorf L., Hale C.J., Stilwell J.L., Yeddula N., Snead K.M. (2023). SGN-B6A: A New Vedotin Antibody-Drug Conjugate Directed to Integrin Beta-6 for Multiple Carcinoma Indications. Mol. Cancer Ther..

[144] Hollebecque A., Lopez J.S., Piha-Paul S.A., Dowlati A., Patnaik A., Galvao V., Bockorny B., Sehgal K., Kingsley E., Sanborn R.E. (2023). SGN-B6A, an integrin beta-6 (ITGB6)-targeted antibody-drug conjugate (ADC), in patients with advanced solid tumors: Updated results from a phase 1 study (SGNB6A-001). J. Clin. Oncol..

[145] Avincsal M.O., Kamizaki K., Jimbo N., Shinomiya H., Nibu K.-I., Nishita M., Minami Y. (2021). Oncogenic E6 and/or E7 proteins drive proliferation and invasion of human papilloma virus-positive head and neck squamous cell cancer through upregulation of Ror2 expression. Oncol. Rep..

[146] Chang H.W., Frey G., Wang J., Liu H., Xing C., Chen J., Boyle W.J., Short J.M. (2025). Preclinical development of ozuriftamab vedotin (BA3021), a novel ROR2-specific conditionally active biologic antibody-drug conjugate. MAbs.

[147] B.H.P. Inc (2022). A Study to Evaluate BA3021 in Participants with Solid Tumors.

[148] Keeling J., Barsh E.R., Melchor N., Aysola K., Weight R.M., Falchook G.S. (2025). First report of ROR2 directed therapy with a conditionally active antibody drug conjugate in advanced melanoma. J. Clin. Oncol..

[149] Wong W., Adkins D., Misleh J., Lorch J., Grewal J., Russell J., Cetnar J., Ho A., Chen K., Aysola K. (2024). 868P Phase II trial of ozuriftamab vedotin (BA3021), a conditionally active biologic (CAB)-ROR2-ADC, in patients with recurrent or metastatic squamous cell carcinoma of the head and neck (R/M SCCHN). Ann. Oncol..

[150] Llop S., Jiménez-Labaig P., Micheletto I., Saba N.F., O’LEary B. (2025). Novel Treatment Approaches in Recurrent/Metastatic Squamous Head and Neck Cancer. Otolaryngol. Clin. N. Am..

[151] Hanna G.J., Lizotte P., Cavanaugh M., Kuo F.C., Shivdasani P., Frieden A., Chau N.G., Schoenfeld J.D., Lorch J.H., Uppaluri R. (2018). Frameshift events predict anti-PD-1/L1 response in head and neck cancer. J. Clin. Investig. Insight.

[152] Saxena M., van der Burg S.H., Melief C.J.M., Bhardwaj N. (2021). Therapeutic cancer vaccines. Nat. Rev. Cancer.

[153] Zeng Q., Zhang S., Leng N., Xing Y. (2024). Advancing tumor vaccines: Overcoming TME challenges, delivery strategies, and biomaterial-based vaccine for enhanced immunotherapy. Crit. Rev. Oncol..

[154] Fernandez E., Vernet R., Urwyler M., Von Rohr O., Charrier E., Belkouch M.-C., Saingier V., Courtout F., DeVito C., Ancrenaz V. (2025). Overall survival of recurrent/metastatic head & neck squamous cell carcinoma patients progressing after ≥1 line of systemic therapy, treated with MVX-ONCO-1, a novel, first-in-class cell encapsulation-based immunotherapy: Results of SAKK 11/16, a phase IIa trial. Exp. Hematol. Oncol..

[155] Vernet R., Fernandez E., Migliorini D., Ancrenaz V., Charrier E., Belkouch M.-C., Von Rohr O., Urwyler M., De Vito C., Renaux J. (2024). A First-in-Human Phase I Clinical Study with MVX-ONCO-1, a Personalized Active Immunotherapy, in Patients with Advanced Solid Tumors. Cancer Res. Commun..

[156] Kenter G.G., Welters M.J.P., Valentijn A.R.P.M., Löwik M.J.G., Berends-van der Meer D.M.A., Vloon A.P.G., Drijfhout J.W., Wafelman A.R., Oostendorp J., Fleuren G.J. (2008). Phase I immunotherapeutic trial with long peptides spanning the E6 and E7 sequences of high-risk human papillomavirus 16 in end-stage cervical cancer patients shows low toxicity and robust immunogenicity. Clin. Cancer Res..

[157] Zwaveling S., Mota S.C.F., Nouta J., Johnson M., Lipford G.B., Offringa R., van der Burg S.H., Melief C.J.M. (2002). Established human papillomavirus type 16-expressing tumors are effectively eradicated following vaccination with long peptides. J. Immunol..

[158] Staff C.N.E. (2021). FDA Gives ISA101b Fast Track Designation for HPV 16+ Oropharyngeal Cancer. https://www.cancernetwork.com/view/fda-gives-isa101b-fast-track-designation-for-hpv-16-oropharyngeal-cancer.

[159] de Sousa L.G., Rajapakshe K., Canales J.R., Chin R.L., Feng L., Wang Q., Barrese T.Z., Massarelli E., William W., Johnson F.M. (2022). ISA101 and nivolumab for HPV-16^+^ cancer: Updated clinical efficacy and immune correlates of response. J. Immunother. Cancer.

[160] Kong A., Even C., Aguilera B., Hesselink M.K., Visscher S., Chung C., Park J., Adkins D., Salas S., Harrington K. (2024). 878P Final results of a phase II study of peltopepimut-S and cemiplimab in patients with relapsed/metastatic HPV16+ oropharyngeal cancer that progressed with prior anti-PD-1 therapy. Ann. Oncol..

[161] Kong A.H., Hesselink M.S.K., Aguilera B., Adkins D., Even C., Fayette J., Muzaffar J., Visscher S., Melief C.J.M., Hooftman L.W. (2023). Phase 2 study of ISA101b (peltopeimut-S) and cemiplimab in patients with advanced HPV16+ oropharyngeal cancer who failed anti-PD-1 therapy. J. Clin. Oncol..

[162] Quayle S.N., Girgis N., Thapa D.R., Merazga Z., Kemp M.M., Histed A., Zhao F., Moreta M., Ruthardt P., Hulot S. (2020). CUE-101, a Novel E7-pHLA-IL2-Fc Fusion Protein, Enhances Tumor Antigen-Specific T-Cell Activation for the Treatment of HPV16-Driven Malignancies. Clin. Cancer Res..

[163] Chung C., Colevas A.D., Gibson M., Adkins D., Sukari A., Wirth L., Burtness B., Bauman J., Rodriguez C., Worden F. (2021). A phase 1 trial of CUE-101, a novel HPV16 E7-pHLA-IL2-Fc fusion protein, alone and in combination with pembrolizumab in patients with recurrent/metastatic HPV16+ head and neck cancer. J. Immunother. Cancer.

[164] Chung C.H., Colevas A.D.D., Adkins D., Rodriguez C.P., Park J.C., Gibson M.K., Burtness B., Johnson F.M., Julian R.A., Saba N.F. (2023). A phase 1 dose-escalation and expansion study of CUE-101, a novel HPV16 E7-pHLA-IL2-Fc fusion protein, given as monotherapy and in combination with pembrolizumab in patients with recurrent/metastatic HPV16+ head and neck cancer. J. Clin. Oncol..

